# Research on the development methodology for clinical practice guidelines for organic integration of traditional Chinese and Western medicine

**DOI:** 10.1186/s40779-023-00481-9

**Published:** 2023-09-27

**Authors:** Ying-Hui Jin, Yan-Ping Wang, Ying-Lan Xie, Gui-Hua Tian, Xiao-Yu Zhang, Nan-Nan Shi, Ke-Hu Yang, Xin Sun, Yao-Long Chen, Da-Rong Wu, Xin-Feng Guo, Long Ge, Chen Zhao, Cheng Lu, Yin Jiang, Jing Guo, Si-Yu Yan, Yong-Bo Wang, Qiao Huang, Xiang-Ying Ren, Ying-Yue Rao, Yun-Yun Wang, Meng-Qian Yuan, Xian-Tao Zeng, Hong-Cai Shang

**Affiliations:** 1 Center for Evidence-Based and Translational Medicine, Zhongnan Hospital of Wuhan University, Wuhan, 430071 China; 2 Institute of Basic Research in Clinical Medicine, China Academy of Chinese Medical Sciences, Beijing, 100700 China; 3 China Center for Evidence Based Traditional Chinese Medicine, Beijing, 100029 China; 4 Hubei Provincial Hospital of Traditional Chinese Medicine, Wuhan, 430074 China; 5 Key Laboratory of Chinese Internal Medicine of Ministry of Education and Beijing, Dongzhimen Hospital, Beijing University of Chinese Medicine, Beijing, China; 6 Evidence-Based Medicine Center, School of Basic Medical Sciences, Lanzhou University, Lanzhou, 730000 China; 7 China Center for Evidence-Based Medicine, West China Hospital of Sichuan University, Chengdu, 610041 China; 8 State Key Laboratory of Dampness Syndrome of Chinese Medicine, The Second Affiliated Hospital of Guangzhou University of Chinese Medicine, Guangzhou, 510120 China; 9 Evidence-based Medicine Team, Guangdong Provincial Hospital of Traditional Chinese Medicine, Guangzhou, 510120 China; 10 Nanjing University of Chinese Medicine, Nanjing, 210023 China; 11 Jiangsu Province Hospital of Chinese Medicine, Nanjing, 210029 China

**Keywords:** Methodology, Traditional Chinese medicine, Western medicine, Organic integration, Clinical practice guidelines

## Abstract

Integrated traditional Chinese medicine (TCM) and Western medicine (WM) is a new medical science grounded in the knowledge bases of both TCM and WM, which then forms a unique modern medical system in China. Integrated TCM and WM has a long history in China, and has made important achievements in the process of clinical diagnosis and treatment. However, the methodological defects in currently published clinical practice guidelines limit its development. The organic integration of TCM and WM is a deeper integration of TCM and WM. To realize the progression of "integration" to "organic integration", a targeted and standardized guideline development methodology is needed. Therefore, the purpose of this study is to establish a standardized development procedure for clinical practice guidelines for the organic integration of TCM and WM to promote the systematic integration of TCM and WM research results into clinical practice guidelines in order to achieve optimal results as the whole is greater than the sum of the parts.

**Supplementary Information:**

The online version contains supplementary material available at 10.1186/s40779-023-00481-9.

## Background

Integrated traditional Chinese and Western medicine is unique to China, and it is also a major feature and advantage of China's medical and health system. The synthesis of integrated traditional Chinese medicine (TCM) with the knowledge of Western medicine (WM) provides the Chinese people with a powerful medical and healthcare system with "Chinese characteristics" [1, 2].

In 2022, the General Office of the State Council of China issued the “14th Five-Year Plan” for the Development of TCM, which clearly named a number of integrated TCM and WM diagnosis and treatment programs that can reflect the "appropriate Chinese and Western medicine" [3]. Accelerating the research and development of clinical practice guidelines (guidelines for short) for integrated TCM and WM, and fully exploiting the advantages of TCM, WM and integrated TCM and WM in the process of disease diagnosis and treatment are important links in promoting TCM to the world, building a community with a shared future for mankind and contributing to the "Chinese plan" [4].

In the past 5 years, more than 50 guidelines or expert consensus statements on integrated TCM and WM have been published each year. Researches show that these still have many methodological defects [5], and the implementation rate is low [6, 7]. There are obvious problems of "knot without compatibility" in the clinical practice guidelines for integrated TCM and WM. The recommendations of TCM and WM are mostly independent, inconsistent with the actual clinical diagnoses and treatment processes, and lacking in important information (for example, at which stage of the disease integrated TCM and WM is better than simple TCM or WM alone? How should they be combined? What are the potential interactions? etc.). All the above issues lead to low operability of the integrated TCM and WM clinical practice guidelines which in turn affects the implementation and transformation of recommendations.

Integrated TCM and WM focuses on integrating the advantages of TCM with WM and avoiding their disadvantages, reflecting the combination of a holistic view and systems biology, individualization and precision medicine. The discipline of integrated TCM and WM uses modern language to clarify the scientific connotation of TCM, and provides the possibility for the evidence-based optimization of "standardized production of evidence" and "accurate differentiation of syndromes", as well as the language translation of organic integration of TCM and WM and the mutual recognition of results [1, 8, 9]. The guidelines for integrated TCM and WM should not be a simple superposition or piling up of pieces of evidence and recommendations of WM and TCM, but a deep organic integration of the advantages of both TCM and WM based on clinical diagnosis and treatment pathways. However, the current guidelines that fit the diagnosis and treatment practices of integrated TCM and WM and can guide the development of full integration of their recommendations are lacking in sound methodology. The existing methodology system cannot support the research and development of guidelines for integrated TCM and WM [10].

A standard research group was set up, consisting of 17 members from the following centers: Center for Evidence-Based and Translational Medicine, Zhongnan Hospital of Wuhan University (Center for Evidence-Based and Translational Medicine, Wuhan University), Dongzhimen Hospital, Beijing University of Chinese Medicine, Institute of Basic Research in Clinical Medicine, China Academy of Chinese Medical Sciences, Hubei Province TCM Hospital, Center for Evidence-Based Medicine, Lanzhou University, The Chinese Cochrane Center, West China Hospital of Sichuan University, Guangdong Province TCM Hospital. This research team approved the "methodologies and procedures for developing clinical practice guidelines of organic integration TCM and WM" in the China Standardization Association, aiming to standardize the development process of guidelines for integrated TCM and WM, and develop guidelines that can reflect its diagnosis and treatment practice and solve its clinical problems, so promoting the systematic integration of TCM and WM research results into guidelines to achieve optimal results as the whole is greater than the sum of the parts.

## Research methods

### Systematic review of the literature

Inclusion criteria: The full text of the guidelines for integrated TCM and WM published at home and abroad was obtained. Among the titles, "integrated TCM and WM (integrative TCM and WM)" and/or "Combination of WM diseases and TCM syndrome differentiation" words or titles include the proper terms of TCM and WM diagnosis and treatment. For example, "Guidelines for combining TCM with antibiotics to treat diseases". In addition, although the titles of some articles are the integration of TCM and WM, the recommendations only cover TCM, which does not meet the requirements, and such articles need to be excluded.

Search strategy: The databases used included PubMed, Web of Science, CNKI, Wanfang Data and VIP database, and the search time limit was from inception to July 2022. The search was carried out using a combination of subject words and free words and adjusted according to the characteristics of each database. References from the included studies were also searched to track the literature that met the inclusion criteria. The Chinese search terms are traditional Chinese medicine, Western medicine, integrated traditional Chinese and Western medicine, Combination of Western medicine diseases and traditional Chinese medicine syndrome differentiation, guidelines, clinical practice guidelines, recommendations, etc. The English search terms are as follows: traditional Chinese medicine, TCM, Western medicine, integrative medicine, integrated traditional Chinese and Western medicine, guide*, clinical practice guideline, recommend*.

Data extraction and screening: Two researchers independently screened the literature, extracted the data, and cross-checked it. Disagreements were resolved through discussion or consultation with a third party. Data extraction included: first author, year of publication, journal of publication, type of disease, whether a population, interventions, comparisons, outcomes (PICO) problem was raised, whether evidence was researched, whether evidence quality evaluation was carried out, whether a suitable model of integrated TCM and WM was proposed, whether recommendation suggestions included TCM and WM diagnosis and treatment methods, whether suggestions of integrated TCM and WM were provided, whether the application of TCM and WM recommended suggestions were clarified, the specific content of the integrated TCM and WM and the application of the relationship.

### Qualitative interviews

A qualitative design with purposive sampling was used to select experts in evidence-based medical methodology and researchers or clinicians who develop clinical practice guidelines for TCM/integrated TCM and WM. The included experts are required to have a doctoral degree, a senior professional title, and at least 10 years of work experience. A semi-structured interview format was used for data collection in an online meeting via Tencent Meeting (a Chinese-based online conference facility). The interviews lasted 40–60 min, and were all completed over a 2 months’ period. Data information saturation was used as a condition for the termination of sample collection.

An outline for the interview was developed from the literature review, including the following questions: What do you think are the methodological or reporting flaws of the existing guidelines for integrated TCM and WM? Do you think that the existing clinical questions in clinical practice guidelines for the integration of TCM and WM are in line with the principles and methods of clinical questioning in guidelines? Do you think that the existing grading system of evidence for TCM is fully applicable to the development of guidelines for integrated TCM and WM? How do you think the quality of evidence of TCM expert opinion/ancient literature can be objectively evaluated? How do you think the guidelines for integrated TCM and WM can fully integrate the recommendations of TCM and WM in order to closely fit the clinical practice of integrated TCM and WM?

NVivo software (version 12, QRS International, Denver, CO, US) was used for data coding and content analysis was used to analyze the data. The themes were refined after joint analysis and discussion by the research team to avoid bias.

### Structure brainstorming

In the standard research group, the professional field covered clinical integrated TCM and WM, methodology of evidence-based medicine (including development and writing of guidelines). The systematic review of previous literature and results of the qualitative research were used as the content of a preliminary discussion for brainstorming. All participants spoke freely and fully on the topic of how to promote the development of guidelines for the organic integration of TCM and WM through methodological guidelines. The research group recorded the opinions of all participants verbatim, and the core research members integrated the information after the meeting by using the recording facility in Tencent Meeting. Based on the information integrated by the above-mentioned methods and steps of guidelines development, "issues to be considered in the guidelines for organic integration of TCM and WM" and "precautions for the guidelines for organic integration of TCM and WM" were developed, so as to improve methodology such as the formulation of clinical problems of the organic integration of TCM and WM, the grading of evidence and the formulation of recommendations, the mode of integrated TCM and WM, the organic integration methods of recommendations, and finally developed the draft of the methodologies and procedures for developing clinical practice guidelines of organic integration of TCM and WM.

### Consensus meeting method

All members of the standard-setting group were invited to discuss, revise, and improve the basic content of the draft through consensus meetings to form the final draft of the methodologies and procedures for developing clinical practice guidelines of organic integration of TCM and WM.

### Delphi expert consultation

A total of 23 multidisciplinary experts from across the country covering the fields of WM, TCM and integrated TCM and WM, evidence-based medicine, pharmacy and nursing were invited to conduct the Delphi consultation. The consultation questionnaires were sent by e-mail and included the terminology, method protocols, diagrams and annexes in the draft. Experts were free to question any content of the exposure draft. Where experts disagreed with the content, they needed to give reasons and specific suggestions for changes. Moreover, the Chinese Society for Standardization conducted a web-based consultation to solicit input from a wide range of developers and users of guidelines for integrated TCM and WM.

### Re-consensus session discussion

The research team collated the returned consultation drafts and a further consensus meeting was held by the standard development team to discuss each standard individually and decide whether to make changes to their content.

### External reviews

**Fig. 1:**
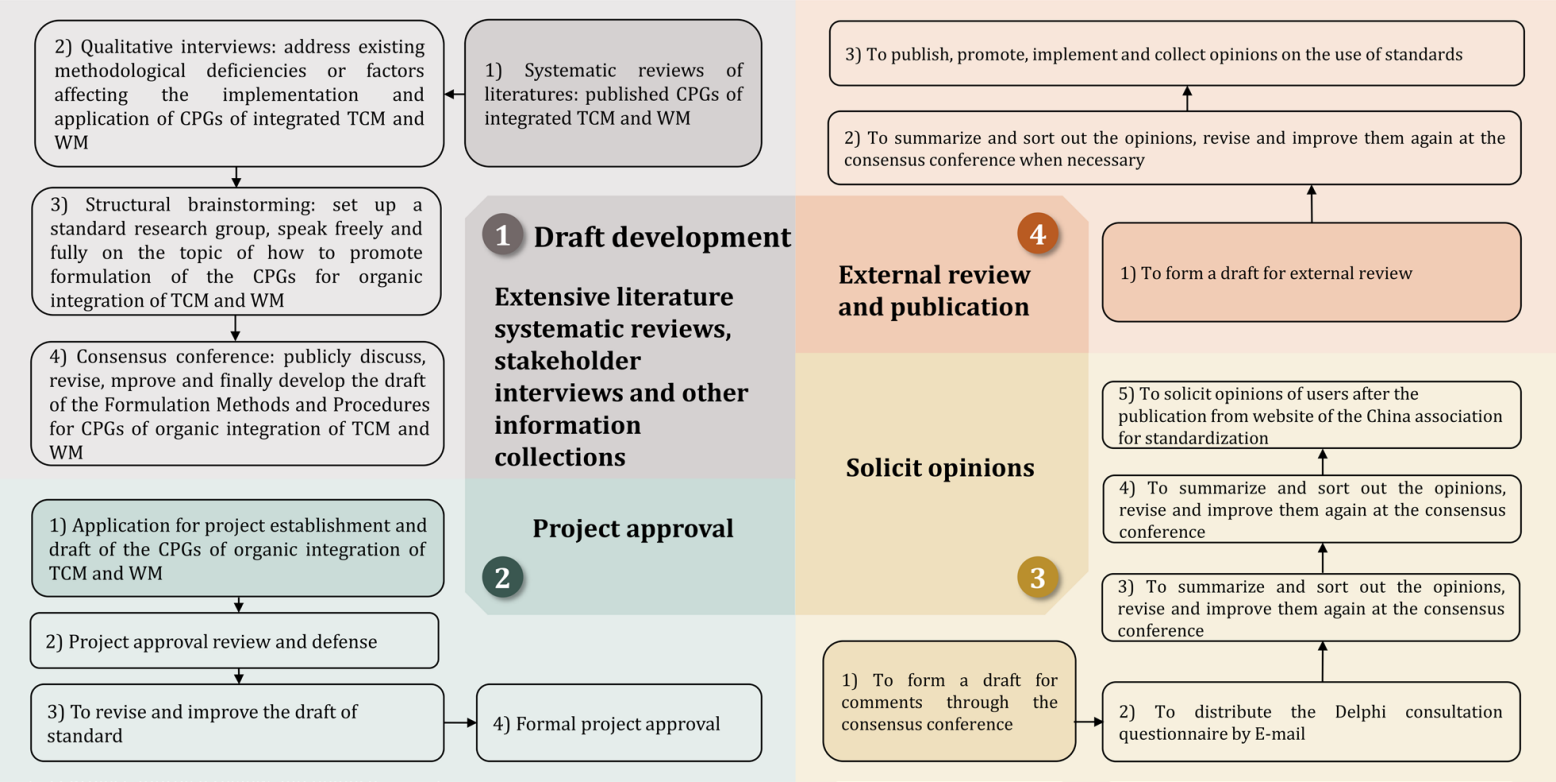
Methodological flowchart for standard of developing guidelines for organic integration of TCM and WM. TCM traditional Chinese medicine, WM Western medicine

Guidelines developers and methodologists in the field of integrated TCM and WM who had not participated in the development and consultation of this standard were then invited to participate in an external review. The review comments were summarized and discussed to make the necessary changes and improvements, and the standard was then published. The methodological flow chart is shown in Fig. 1.

## Results

### Systematic review of the clinical practice guidelines for integrated TCM and WM

A search obtained 54 guidelines for integrated TCM and WM. Only 3 (5.6%) of the included studies clearly presented clinical problems based on the idea of integrated TCM and WM. Only 26 (48.1%) papers described how to use the TCM and WM treatments to provide clinical ideas related to integrated TCM and WM, i.e., analyzing why TCM and WM can be integrated, and the advantages and disadvantages of each side. Only 22 (40.7%) papers gave the relationship between integrated TCM and WM, i.e., they specified how to use the treatment methods of TCM and WM in different situations (stage, staging, classification or syndrome differentiation) and how to make trade-offs, and indicated the order of priority and preference between TCM and WM treatments, including the combined use, recommendation of TCM, recommendation of WM, no recommendation of TCM, and no recommendation of WM. In the remaining guidelines TCM and WM recommendations are given separately, and the recommended intervention protocols are not sufficiently qualified to determine the relationship between the TCM and WM recommended intervention protocols, and give limited guidelines.

### The findings of the qualitative research

A total of 20 experts in evidence-based medical methodology, researchers or clinicians with experience in developing guidelines in TCM/integration of TCM and WM were interviewed. The analysis yielded six themes and twelve sub-themes, as shown in Table 1.

**Table 1:** Themes and sub-themes of qualitative research

Themes	Sub-themes
The problems of "incompatibility" are highlighted	The standardized development, dissemination and implementation of clinical practice guidelines of organic integration of TCM and WM is the only way for the real organic integration to be achieved The current situation is a simple superposition or stack of evidence and recommendations of WM and TCM
Objectives of organic integration of TCM and WM	The organic integration of TCM and WM is based on integrated TCM and WM, but goes further and lays more emphasis on the complementary advantages of TCM and WM in the interpretation of mechanisms, clinical research and clinical practice The guideline for organic integration of TCM and WM should not be a simple superposition or stack of evidence and recommendations of WM and TCM. It is the deep organic integration of the advantages of TCM and WM based on the clinical diagnosis and treatment pathways
Presentation of clinical problems in Clinical practice guidelines of organic integration of TCM and WM	The presentation of clinical problems in the development of clinical practice guidelines for integration of TCM and WM does not reflect the problems that urgently need to be solved in the process of integrated TCM and WM diagnosis and treatment practice, so the guidelines are not instructive to clinical practice The clinical problems were not specific and clear enough to guide the literature search at the later stage
Selection and presentation of GQESRs in the guideline	Controversy over use of different GQESRs within a single guideline Paying attention to clear and understandable labeling systems for two grading systems
Traditional evidence for TCM**–** ancient literature	Controversy over the use of ancient literature as evidence in the guideline Multi-dimensional evaluation of evidence from ancient literature
Traditional evidence for TCM**–** expert experience	Confirmed expert experience used as evidence in the guideline Multi-dimensional evaluation of evidence from expert experience

*GQESR* Grading Quality of Evidence and Strength of Recommendations, *TCM* traditional Chinese medicine, *WM* Western medicine

### The concept modification of clinical practice guidelines for “organic integration of TCM and WM”

In the internal discussion of the project team, researcher Hong-Cai Shang pointed out that TCM and WM should not only be integrated, but also go further to achieve organic integration of TCM and WM. "Integration" refers to the superficial connection. Which often involves "incompatibility", "organic integration" means that materials enter each other and become one. Organic integration is really the combination and integration of TCM and WM to form a new system. The guidelines for organic integration of TCM and WM should not be a simple superposition or collection of evidence and recommendations of both WM and TCM. It is the deep organic integration of the advantages of TCM and WM based on the clinical diagnosis and treatment pathways. Therefore, after repeated discussion, this standard has been named "methodologies and procedures for developing clinical practice guidelines for organic integration of traditional Chinese and Western medicine".

### The formation of the structure for developing methods and procedures for clinical practice guidelines for organic integration of TCM and WM

Starting from each step of guideline development, "Specific conceptual problems of clinical practice guidelines for organic integration of TCM and WM" were introduced by asking questions, to guide and standardize the process of guideline development for organic integration of TCM and WM, and concurrently develop the "notes" in the guideline steps see Additional file 1. Most of these notes are aimed at the unique methodological points of integrated TCM and WM to prompt the developers of the guidelines to read and follow them.

#### Questions needing consideration regarding organic integration of TCM and WM

(1) Whether there is a relationship between WM diseases and TCM diseases or a relationship between WM diseases and TCM syndrome differentiation and whether the scope of integrated TCM and WM involved in the theme of the guidelines for organic integration TCM and WM has been determined through extensive professional consensus when selecting the guideline theme?

(2) According to the guidelines theme determined in question 1, it should be further determined whether the guideline is named using WM disease names, TCM disease names or TCM syndrome differentiation.

(3) Does the practice of diagnosis and treatment of the disease fully reflect the characteristics of organic integration of TCM and WM? Instead of simply focusing on TCM or WM, or simply adding the two. For example, when treating a viral cold or a certain stage of the cold’s development, a certain sub-type, a certain syndrome or a certain symptom of a viral cold, some Chinese medicine or traditional Chinese herbs may have the effect of replacing and/or supplementing WM, while some may exert attenuating and/or enhancing effects on WM or other WM therapies; and vice versa.

#### Questions needing consideration about the establishing question of clinical practice guidelines for organic integration of TCM and WM

(1) Does the proposal of the problem come from the practice of integrated TCM and WM, and include the idea of integrated TCM and WM treatment?

(2) Are TCM clinicians and WM clinicians, as well as clinicians combining Chinese and Western medicine all selected for the questionnaire?

(3) Guideline developers should give full consideration to the characteristics of combined treatment of diseases with TCM and WM treatment and include specific outcomes for TCM before ranking the outcomes’ importance.

#### Points for consideration when contemplating proposal writing for the clinical practice guidelines for organic integration of TCM and WM

Do the guidelines explicitly use integrated TCM and WM models of diagnosis and treatment? (For example, "combination of WM diseases and TCM syndrome differentiation", "combination of WM and TCM at different stages of disease", "syndrome differentiation and targeted therapy", and "multidisciplinary diagnosis and treatment").

#### Questions to be considered in response to the evidence search for clinical practice guidelines for organic integration of TCM and WM

(1) Has there been a complete retrieval of evidence sources for TCM, WM and integrated TCM and WM (such as consulting experts in the field of TCM, searching the featured TCM database)?

(2) Has there been a collection of characteristic evidence of TCM, such as TCM ancient books and expert experience?

(3) Has there been a collection of evidence of patients' preferences, willingness, and values for accepting TCM or WM treatments?

(4) Besides the above considerations, has there been a complete retrieval of information on the "body of evidence" for a specific clinical question? First, the "body of evidence" can be derived from a synthesis of pieces of evidence from the best research design, or it can be composed of evidence from multiple research methods and multiple sources. For example, when real-world evidence exists, ancient documentary evidence and expert opinion evidence can be corroborated with real-world evidence to form a "body of evidence". Again, for example, the "body of evidence" from randomized controlled trials provides evidence of intervention effectiveness, while studies such as observational studies or the registration and recording platforms of adverse reactions can provide evidence of intervention implementation and safety, and the two types of evidence together support the designation of scientific and rational decision-making.

#### Questions to be considered about the evaluation of the quality of evidence and the classification of recommendations in the clinical practice guidelines for organic integration of TCM and WM

(1) Is it necessary to classify the quality of evidence with TCM characteristics such as TCM ancient books and expert experience? Or is there a custom classification standard of the quality of evidence and recommendations for the guidelines for organic integration of TCM and WM?

(2) How should an appropriate grading system for the quality of evidence and recommendations be chosen in order to achieve a reasonable classification of included TCM and WM literature? In this process, is it necessary to make appropriate adjustments to the existing grading system of the quality of evidence and recommendations (especially the grading system based only on the modern medical system)? Has the adjustment method passed the consensus of the guidelines’ development group?

#### Questions to be considered about the method for forming the recommendations for the clinical practice guidelines for organic integration of TCM and WM

(1) When forming the recommendations for the organic integration of TCM and WM, are clinical experts from TCM, WM and integrated TCM and WM all invited to agree on the recommendations?

(2) Does the generated recommendation explain the relationship between TCM treatment, WM treatment and integrated TCM and WM treatment?

#### Critical questions to be considered in response to the report of clinical practice guidelines for organic integration of TCM and WM

(1) Does the title of the guidelines clearly state "clinical practice guidelines for organic integration of TCM and WM?"

(2) Has a recommendation implementation pathway map or other visual form been developed to present the recommendation, which reflects the full organic integration of TCM and WM recommendations?

(3) Is the report of clinical practice guidelines for organic integration of TCM and WM based on the pre-set integrated TCM and WM model?

### Methodological points and graphic design: analysis and illustration of the relationship between TCM and WM diseases or TCM syndromes involved in the guidelines

The existing guidelines for integrated TCM and WM often use the combined diagnosis of dual disease names of TCM and WM. In TCM and WM combined with double disease diagnosis, WM diagnosis of disease based on WM diagnostic criteria, and then based on the primary symptom of disease using TCM diagnosis, to determine the guidelines contain diseases and disease categories of TCM and WM common to the three kinds of situations. In this standard, the correlation diagram of disease names of organic integration of TCM and WM is designed to show the three common situations: the scope of WM diseases includes the category of TCM disease names, the category of TCM disease names includes a variety of Western diseases and the scope of Western diseases includes a variety of TCM disease names. It is used to guide the guidelines developer to consider the relationship between WM diseases and TCM diseases, and the relationship between WM diseases and TCM syndrome differentiation, so as to rationally determine the theme of the guidelines.

### The relationship between the clinical questions and the guidelines topic (disease/syndrome differentiation) and their mapping

The clinical problems in the guidelines of organic integration of TCM and WM should be generated from the diagnosis and treatment practice of integrated TCM and WM, especially for the most confusing diagnoses and treatment problems for clinicians, and should reflect the characteristics and ideas of integrated TCM and WM diagnosis and treatment.

After the theme of the guideline is determined, the guidelines developer determines the clinical problem, and it is necessary to consider whether the question raised is specific and clear. The relationship between WM diseases, TCM diseases and the corresponding syndrome differentiation or staging types must have been analyzed when determining the theme. Clinical problems need to be raised and reflect the needs of clinical diagnosis and treatment practice after the above relationship is clarified. This standard uses graphics to show PICO problems and the relationship between disease name and syndrome differentiation, which can not only guide the guidelines developer to refine PICO clinical problems, but also help make the clinical problems more specific and clearer.

### Analysis and illustration of evidence source level and structure for the development of clinical practice guidelines for organic integration of TCM and WM

Evidence retrieval for guidelines for organic integration of TCM and WM should reflect a full search of the evidence sources of TCM, WM and integrated TCM and WM, and have considered the retrieval and inclusion of TCM characteristic evidence. In addition, the relationship between multi-source evidence and the development of guidelines should be clarified. This standard, emphasizes the importance of ensuring that ancient TCM books and modern TCM expert experience have also undergone validation and evaluation by clinical research (including traditional clinical research or clinical research based on real-world data), so that it can enter the summary and evaluation of secondary studies and become the evidence source for guidelines. That is, the TCM classical evidence can be inherited through the verification of ancient and modern evidence. We have integrated the above content into a schematic diagram of the evidence source level and structure of the guidelines of organic integration of TCM and WM.

### Design of accessories to this standard

In order to make it easier to refer to this standard, we have designed seven appendices (Additional file 1), which are: The presentation of the clinical practice guidelines for the Organic Integration of TCM and WM; The judgment of problems with clinical practice guidelines for Organic Integration of TCM and WM and the importance of outcome indicators; the integration model of TCM and WM; the statement of conflict of interest; Evidence summary table and consensus voting table; Examples of recommendations which can reflect the intervention relationship between TCM and WM; Outline of the process and steps for developing clinical practice guidelines.

### How to use this standard

When developing guidelines in the field of integrated TCM and WM, guidelines developers can still refer to the basic methodology of other published guidelines (especially in the field of WM) to understand many details of the guidelines’ development process, such as the contributors and their role in guidelines development, literature retrieval and systematic review of the guidelines development team. Moreover, the inclusion of this standard before each step can help guidelines developers to formulate guidelines with full organic integration of TCM and WM recommendations in the practice of diagnosis and treatment of TCM and WM.

Dual diagnosis is often used in the existing guidelines for integrated TCM and WM. In dual diagnosis, the WM disease name is based on Western medical diagnostic criteria, and then the TCM disease name is based on the main symptoms of the disease. Therefore, it can determine the scope of both the Western medical disease and the Chinese medical disease included in the guidelines. In this standard, we have listed three patterns in general and the diagram with examples to help the readers to confirm the appropriate guidelines’ theme and PICO questions. The development of guidelines for organic integration of TCM and WM is often faced with difficulties because of insufficient evidence, especially the lack of high-quality studies on the comparison of integrated TCM and WM and non-integrated TCM and WM. At this point, the guidelines development group needs to gather expert evidence that includes empirical feedback from long-term practice provided by people who are knowledgeable or technically skilled in a particular field on the clinical issues that urgently need to be addressed, and how to use and evaluate evidence from ancient TCM literature and the experience of modern TCM experts. This standard has given detailed suggestions. The guidelines panel needs to read this standard before writing the guideline protocols to make sure the specific issues about guideline methodologies and practice about organic integration of TCM and WM are fully considered. After the guideline is completed, the guidelines panel should also review the standard again to determine the completion of each step, especially regarding clear and operable presentation of organic integration of TCM and WM recommendations.

## Discussion

With the development of evidence-based medicine (EBM), guidelines play an increasingly important role in healthcare decision-making both in China and worldwide. High-quality guidelines development depends on the complete and rigorous guidelines development methodology [11].

It is not difficult to acknowledge the achievements and develop the guidelines for integrated TCM and WM. At the present stage, integrated TCM and WM typically remains at the level of the combination of TCM and WM diagnosis and treatment methods, and the diagnosis and treatment methods of the two modes are relatively separate, and this can't actually guide the clinical diagnosis and treatment practice of TCM and WM. The development of integrated TCM and WM should focus on clinical practice, analyze the relationship between TCM and WM, find the confluence, and seek new concepts and methods of integrating TCM and WM [12]. Origin of Chinese characters records: *"Integration" refers to a superficial connection, which often still has areas of incompatibility. Organic integration means the cooking gas comes out", "Organic Integration" means that materials enter into each other and become one* [13]. Organic Integration is really the addition and integration of TCM and WM to form a new system. Therefore, many scholars now propose that TCM and WM should not only be integrated, but should also achieve organic integration. They believe that the future of integrated TCM and WM is the pathway "from integration to organic integration" [14].

The establishment of this standard is strictly in accordance with the construction rules of the group standard of China Association for Standardization to conduct project approval and development and solicitation of opinions on standards. The development process includes the literature review and expert interview, information extraction, discussion and analysis, collection of opinions from consensus meetings and subsequent revision. Participants in the standard development process include methodologists, clinical experts, and targeted users of the guidelines developed for TCM, WM and integrated TCM and WM. The purpose of this standard is to establish a method for developing guidelines in the field of integrated TCM and WM. The content provides: 1) Basic steps and procedures of guidelines development; 2) The important issues in developing guidelines for organic integration of TCM and WM. At each step, these are presented as "problems that need to be considered" with eye-catching titles, which very easily attract the attention of the guidelines’ developers. This allows them to consider the unique methodological problems of TCM and WM organic integration and helps them to raise clinical problems from the practice of TCM and WM diagnosis and treatment. They can then define the integrated TCM and WM model, so achieving complete evidence grading and rational evaluation of evidence quality. This can, in turn, guide the writing of recommendations and opinions and the construction of diagnosis and treatment procedures based on the integration of TCM and WM in the hope of achieving implementation of the guidelines from their development; 3) It provides the points for attention in the development of various guidelines steps from the perspective of organic integration of TCM and WM, which can enhance their prompting and guiding functions; 4) Seven appendixes are provided for reference and to aid operability. However, this standard still has obvious limitations, one of the main reasons for the low quality of integrated TCM and WM guidelines is the lack of high-quality and convincing evidence that integrated TCM and WM is superior to WM alone. There is less evidence available to carry out controlled studies given the realistic timing of TCM and WM treatment and their respective characteristics and advantages. When the evidence is seriously insufficient it is difficult to form clinical recommendations even with highly operational methodological guidelines.

Establishing a standardized development procedure for guidelines for the organic integration of TCM and WM can provide a feasible method for systematically integrating TCM and WM, promote the communication and cooperation between TCM and WM, and form a comprehensive treatment model for disease diagnosis and treatment.

This standard has specified the method for developing guidelines in the field of integrated TCM and WM, so it is mainly applicable to those who develop these guidelines. We believe that they are not confined to China and not confined to the integration of TCM and WM.

As a genre of traditional medicine, TCM has gained popularity worldwide. At present, more than 180 countries and regions in the world recognize TCM and use TCM to treat diseases to a greater or lesser extent. In the Asian region, TCM is widely used in China, Japan, Korea, Vietnam, Malaysia, and other countries. Moreover, TCM has been gradually recognized and accepted in Europe, America, and other regions. Many countries have begun to introduce TCM into their medical systems, combining it with modern medicine to provide a more comprehensive treatment plan [15]. In 2014, the WHO released a 10-year strategy that aims to integrate traditional medicine into modern medical care to achieve universal health coverage. The document calls on member states to develop healthcare facilities for traditional medicine, to ensure that insurance companies and reimbursement systems consider supporting traditional medicine and to promote education in the practices [16].

Several surveys in the USA, Denmark, Sweden and Norway indicate a high consumption of complementary and alternative medicine (CAM) [17]. An interview study from the National Center for Health Statistics with over 31,000 participants indicated that 36% of American adults had used some form of CAM therapy (self- or practitioner-provided) during the last 12 months [18]. The forms and methods of integration of CAM and WM are similar to those of integration of TCM and WM in clinical practice: both need to consider the integration of time points, methods and forms in achieving effective integration.

For guidelines developers, by including international guidelines developers and guidelines developers in the WM professional field, when collecting and formulating clinical problems, it is possible to encounter clinical questions about the use of integrated TCM and WM or other types of CAM combined with WM in clinical practice. Both guidelines’ developers and health professionals should consider the current stage of the disease and how these modalities should be integrated. What are the potential interactions and so on? This standard can be applied in many ways.

When formulating recommendations, considering the generalization, suitability, and potential resource utilization, while also balancing clinical advantages and disadvantages is quite necessary and helps to form rigorous and reasonable recommendations. This view and method have been considered and practiced in many published guidelines [19, 20]. Considering those affecting factors for developing guidelines for integrated TCM and WM, especially in the selection of intervention measures between TCM and WM, the generalization of intervention and the values of patients may have obvious differences. Guidelines developers can still refer to the methodology handbook of other guidelines developing and academic organizations to get detailed operational methods.

The organic integration of TCM and WM is better at solving clinical problems, such as improving efficacy and detoxification, reducing the drug dosage, early intervention, avoiding surgery, and reducing complications. It is an international trend of future medical development. This standard aims to further promote the organic integration of TCM and WM by developing high-quality guidelines for organic integration of TCM and WM and understanding diseases and life from multiple perspectives and world views. Finally, it promotes integrating and developing Chinese and Western cultural concepts and clinical practice internationally.

### Supplementary Information

**Additional file 1:** The formulation methods and procedures for clinical practice guidelines for organic integration of traditional Chinese and Western medicine.

## Data Availability

Not applicable.

## Abbreviations

CAM: Complementary and alternative medicine
EBMs: Evidence-based medicine
GQESR: Grading Quality of Evidence and Strength of Recommendations
PICO: Population, interventions, comparisons, outcomes
TCM: Traditional Chinese medicine
WM: Western medicine
