# Supplementary material for: Research on the development methodology for clinical practice guidelines for organic integration of traditional Chinese and Western medicine
Source: Mil Med Res. 2023 Sep 27;10:45. doi: 10.1186/s40779-023-00481-9 (PMC10523673; doi:10.1186/s40779-023-00481-9)
Supplement: Supplementary file 1 — Additional file 1: The formulation methods and procedures for clinical practice guidelines for organic integration of traditional Chinese and Western medicine. [file 40779_2023_481_MOESM1_ESM.pdf]

Introduction

Integrated traditional Chinese and Western medicine involves synthesizing the knowledge of traditional Chinese medicine (TCM) with the knowledge of Western medicine (WM). This process amalgamates the essence of the ancient and the modern, TCM and WM, and creates a brand-new medical science in our country, which is an important symbol of achieving the modernization of medical science [1]. The root of integrated TCM and WM can be traced back to Chairman Ze-Dong Mao's series of directives in the last century. Chairman Mao said that we should combine the knowledge of TCM and WM to create a unified new medicine and new pharmacology in China. In 1955, in response to Chairman Mao's call, the Ministry of Health hosted the first national course for WM practitioners to learn TCM. Following this, various provinces and cities in China held a series of national courses for teaching TCM to WM practitioners: the door to integrated TCM and WM was officially opened. In 1981, the first National Congress of integrated TCM and WM was held. In the same year, the Chinese Association for Science and Technology approved the establishment of the Chinese Association of Integrated TCM and WM and the founding of the *Chinese Journal of Integrated Traditional Chinese and Western Medicine* [2].

The integration of TCM and WM has been developing for nearly 70 years, during which time it has not only experienced various challenges but has also achieved extraordinary successes. However, it is evident that although there are achievements in integrated TCM and WM the existing methods mostly stay at the experiential level, or simply involve the addition of certain treatment methods, and this cannot really guide its clinical diagnosis and treatment practices. The development of the diagnosis and treatment methods of integrated TCM and WM needs to focus on clinical practice, seek the convergence of TCM and WM relating to the clinical problems, and apply evidence-based methods as a breakthrough, to deeply explore the mechanism, form and level of the combination of TCM and WM in the practice of diagnosis and treatment.

"Organic integration of TCM and WM" is the development and deepening of the connotation of "integrated TCM and WM". Many scholars have proposed that TCM and WM should not only be integrated, but should also achieve organic integration. They believe that the

future of integration of TCM and WM is the pathway "from integration to organic integration" [3].

To realize the progression of "integration" of TCM and WM to "organic integration", the first thing is to formulate complete and standardized clinical practice guidelines (guidelines for short) for the organic integration of TCM and WM. This should not be a simple superposition or piling up of evidence and recommendations of WM and TCM, but a deep organic integration of the advantages of applying both TCM and WM on the basis of clinical diagnosis and treatment pathways. However, at present, a problem of "incompatibility" in the guidelines for integrated TCM and WM remains. The recommendations of TCM and WM are mostly independent, inconsistent with the actual clinical diagnosis and treatment process and lacking important information, leading to low operability of the guidelines which means that they cannot be implemented in clinical practice, limiting the development of organic integration of TCM and WM. The standardized formulation, dissemination and implementation of guidelines for organic integration of TCM and WM is the only way for the real organic integration to be achieved [4].

The formulation methods and procedures for clinical practice guidelines for organic integration of traditional Chinese and Western medicine

1 Scope

This standard specifies the method for formulating guidelines in the field of integrated TCM and WM. It gives suggestions for the formation of development teams for integrated TCM and WM, covers both, TCM and WM guidelines, including standards for the chairman, the guidelines committee, the guidelines development team (including the system evaluation team, the guidelines consensus team), the external audit team and other personnel, and is applicable to various clinical specialties and methodological personnel involved in guideline development.

This standard applies to both original guidelines and updated guidelines, but not to the introduction and adaptation of guidelines.

2 Normative references

The following documents are indispensable for the application of this document. For dated references, the dated version only applies to this document. For undated references, the latest version (including all amendments) applies to this document.

There are no normative references in this document.

3 Terms and definitions

The following terms and definitions apply to this document.

3.1 Clinical practice guidelines (CPGs)

CPGs propose the optimal guidelines related to patients' specific clinical problems, based on the results of a systematic evaluation of available evidence, after a comprehensive analysis of the pros and cons of various alternative interventions.

At present, it is normal practice that evidence-based medicine methodology is based on comprehensive retrieval and strict evaluation of evidence, taking into account the patient's willingness and value preference, resource consumption and other factors.

Guidelines are then formulated using the same standardized and scientific method, which is the main method for formulating international and domestic guidelines. Domestic guideline developers often divide the guidelines into clinical practice guidelines and expert consensus. Some researchers tend to think that expert consensus is an occupational guidance document of lower quality and influence than guidelines, that is, the guidelines are more scientific, transparent and reliable. This standard does not define the above classifications here, and it is suggested that the guidelines and expert consensus should both be developed in accordance with the guidelines for the standard.

3.2 Integration

Origin of Chinese Characters records: "Integration" refers to a superficial connection, which often still has areas of incompatibility.

In *Oxford Dictionary of English*, "integrate" refers to the combination (one thing) with another to form a whole.

3.3 Organic integration

Origin of Chinese Characters records: Organic integration means the cooking gas comes out", "Organic Integration" means that materials enter into each other and become one. Organic integration is really the addition and integration of TCM and WM to form a new system.

In *Oxford Dictionary of English*, "organic" means "denoting or characterized by a harmonious relationship between the elements of a whole".

3.4 Clinical practice guidelines for organic integration of TCM and WM

Integrated TCM and WM is a comprehensive medical discipline based on the ontology and epistemology of TCM, as well as the reductionism, systematology, information theory and cybernetics of modern medicine. It studies the phenomenon, nature and timely evolution of human life and disease, promotes human health, and prevents and treats diseases. The organic integration of TCM and WM is based on the integration of TCM and WM, but goes further and lays more emphasis on the complementary

advantages of TCM and WM in the interpretation of mechanisms, clinical research and clinical practice.

Guidelines for the organic integration of TCM and WM are guidelines that provide TCM doctors, WM and integrated TCM and WM doctors with recommendations on TCM and WM modalities. Generally, the topics of these guidelines are oriented towards the diagnosis and treatment of dominant diseases found in both TCM and WM. They aim to solve the problem of how to choose the intervention measures of TCM and WM in clinical practice. The guidelines for organic integration of TCM and WM should not be a simple superposition or stack of evidence and recommendations of WM and TCM. It is the deep organic integration of the advantages of TCM and WM based on the clinical diagnosis and treatment pathways.

Except for the word "organic integration of TCM and WM" which has been used in the newly proposed "Clinical practice guidelines for organic integration of TCM and WM" in this standard, other previous guidelines in the field of integrated TCM and WM or words related to the discipline of integrated TCM and WM have all used the word "Integrated TCM and WM".

3.5 Conflict of interest

As for the difference between personal interests and professional obligations, an independent third party has reason to question whether an individual's professional behavior or decision is driven by personal interests (such as financial, academic, clinical income or social status), economic or professional relationships.

4 Requirements

4.1 General

The guidelines for organic integration of TCM and WM should focus on clinical practice, seek the combination of TCM and WM from clinical problems, utilize evidence-based methods as the way forward, have as the goal the full integration of the recommendations of TCM and WM, and achieve the full integration of TCM and WM [5]. The guidelines should meet the following requirements in the process of

formulation.

4.2 Determination of guidelines theme

4.2.1 General principles or methods

The first step in formulating a guideline is to establish a theme, that is, to clarify the purpose, significance and scope of application of the guideline. Generally speaking, the guideline consists of one "theme" and several "clinical problems". Clinical problems are the basis for formulating literature retrieval strategies and systematic evaluation and finally forming recommendations. The determination of the guideline theme mainly considers the following factors: 1) The theme has important clinical significance, such as diseases having a high incidence rate, morbidity or mortality or posing a heavy economic burden, and the implementation of the guideline is likely to improve important patient outcomes and reduce medical costs. 2) The subject presents with large differences in clinical practice or clinical research results. 3) At present, there are no existing effective guidelines on relevant topics for use. 4) The research evidence is relatively sufficient; 5) The selected guideline theme is one of the "Dominant diseases" of integrated TCM and WM? "Dominant diseases" refer to diseases with "comparative advantages" in the efficacy, safety, compliance and other aspects of integrated TCM and WM.

4.2.2 For the determination of the theme of the clinical practice guidelines for organic integration of TCM and WM, the following questions should be considered:

Whether there is a relationship between WM diseases and TCM diseases or a relationship between WM diseases and TCM syndrome differentiation and whether, when selecting the guideline theme, the scope of integrated TCM and WM involved in the theme of the guidelines for organic integration TCM and WM has been determined through extensive professional consensus?

According to the guideline theme determined in question (1), it should be further determined whether the guideline is named by WM disease names, TCM disease names or TCM syndrome differentiation.

Does the practice of diagnosis and treatment of the disease fully reflect the characteristics of organic integration of TCM and WM? Instead of simply focusing on TCM or WM, or simply adding the two. For example, for a viral cold or a certain stage, a certain subtype, a certain syndrome or a certain symptom of a viral cold, some Chinese medicine/traditional Chinese herbs may have the effect of replacing and/or supplementing WM, while some may exert attenuated and/or enhancing effects on WM or other WM therapies; and vice versa.

4.2.3 Considerations for the development of clinical practice guidelines for organic integration of TCM and WM

Dual diagnosis is often used in the existing guidelines for integrated TCM and WM. In dual diagnosis, the WM disease name is based on Western medical diagnostic criteria, and then the TCM disease name is based on the main symptoms of the disease. Therefore, it can determine the scope of both the Western medical disease and the Chinese medical disease included in the guidelines. There are three patterns in general (**Fig. S1**). In the following diagram and examples, the pattern in **Fig. S1a** shows that Western diseases are included in the scope of TCM disease names. For example, knee osteoarthritis (KOA) falls under the category of "knee paralysis" in Chinese medicine. The pattern in **Fig. S1b** shows that different diseases share common pathogenesis at different stages of development and can be diagnosed according to TCM pathogenesis in conjunction with WM disease names. For example, "blood stasis syndrome" is seen in the context of coronary heart disease, cerebral infarction, etc. The pattern in **Fig. S1c** shows that when the main symptoms of the disease are different at different stages of disease development, the range of WM diseases can involve multiple TCM disease categories. For example, diabetes mellitus falls under the Chinese medicine categories of "thirst disorders" and "spleen disease". For another example, IgA nephropathy does not have a specific Chinese medicine name and is mostly classified as "hematuria", "edema", "lumbago", "kidney wind", "consumptive disease", and other categories.

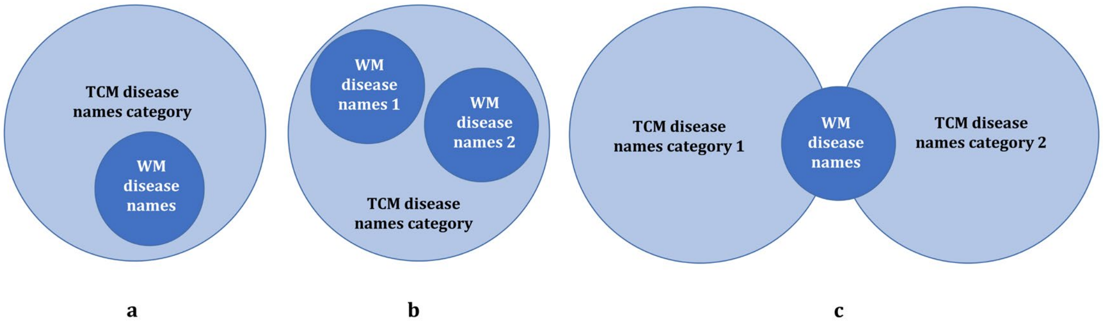

Fig. S1 Diagram of the disease name relationship between organic integration of TCM and WM**. a** The scope of WM disease names is included in the category of TCM disease names category. **b** The scope of TCM disease names category includes a variety of WM diseases; **c** The scope of WM diseases involves a number of TCM disease names category. TCM traditional Chinese medicine, WM western medicine

4.3 Identification of clinical issues for guidelines

4.3.1 General principles or methods

The clinical question is the basis for the final recommendation. The method of constructing the questions can be guided by the current international model: the PICOS model. P is the specific population, which mainly defines the target population and specifies which characteristics are to be considered; I (or E) is the intervention or exposure, which may also be a prognostic factor or trial break; C is the control or another intervention that can be used for comparison; O is the outcome, which describes the outcome of interest; S is the study design, which defines which type of study design is used. A guideline often contains multiple PICOS questions. Identifying clinical questions for guidelines requires systematic literature reviews, qualitative interviews, and questionnaires with stakeholders. Questionnaires need to be broadly representative and therefore must take full account of the geographical distribution of respondents, and multidisciplinary balance (also, the gender or ethnic balance, where relevant). It is recommended that a stakeholder analysis be conducted before the interviews or research to identify the population that the guidelines are likely to involve or affect, to hear and obtain a wide range of stakeholder views before asking questions, selecting and ranking the outcomes.

4.3.2 Regarding the question of establishing clinical practice guidelines for organic integration of TCM and WM, the following questions should be considered:

Does the proposal of the problem come from the practice of integrated TCM and WM, and include the idea of integrated TCM and WM treatment? Refer to **Appendix A** (Example of clinical question formulation)?

Are TCM clinicians and WM clinicians, as well as clinicians combining Chinese and Western medicine all selected for the questionnaire? Refer to **Appendix B** (Template for a basic framework for research on clinical questions and outcome indicators)?

Guideline developers should fully consider the characteristics of combined treatment of diseases with TCM and WM treatment and include a specific outcome for TCM before ranking the outcomes importance.

4.3.3 Considerations for the development of clinical practice guidelines for organic integration of TCM and WM

The question formulation (**Fig. S2**) for the guidelines for organic integration of TCM and WM should be generated from the practice of integrating TCM and WM, especially for the most confusing problems faced by clinicians, and should reflect the characteristics and ideas related to the integration of Chinese and Western medicine.

The clinical questions identified should be specific and clear, and can function as a direct guide for the subsequent literature search. Clinical questions like "whether the combination of proprietary Chinese medicine with conventional Western medicine is better than conventional Western medicine alone in the treatment of a disease" are too broad.

Clinical questions for Chinese patent medicine should be identified in addition to the clinician research mentioned above, the clinical questionnaire should also be refined by extensive searches of Chinese patent medicine codex books, national medical insurance catalogues and basic drug catalogues. Searches of the official State Drug Administration website to identify certain Chinese patent medicine that are legally marketed in mainland China are needed, and discontinued drugs should be eliminated.

Questionnaire research on the use of Chinese patent medicine in clinical practice is needed to further explore the importance of clinical problems based on the use of certain proprietary Chinese medicine alone or in combination with Western medicine.

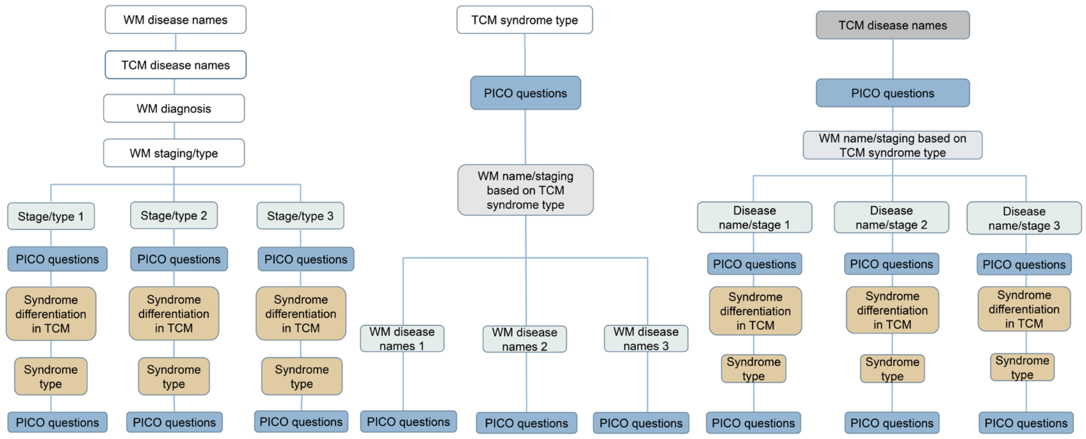

Fig. S2 Example of the relationship between clinical question formulation and guideline topic (disease/syndrome differentiation). *The "TCM disease names" in the diagram on the left are shown as dashed boxes, as it is often not possible to match the WM names with the TCM disease names. TCM traditional Chinese medicine, WM western medicine, PICO population, intervention, control or comparison, outcome.

4.4 Writing and registration of guideline protocols

4.4.1 General principles or methods

A Guideline Proposal/Protocol is a summary document that outlines a plan or series of steps showing how the guidelines will be developed, including all proposed methods. The proposal generally includes background, the institution, and organization developing or involved with it, the target population, the intended end users of the guideline, the composition and roles of the development team, the topic, purpose, scope and clinical issues. It should also show how evidence was obtained, how recommendations were generated, any external review and publication strategy, the timeline of key steps, funding and access to funding, conflict of interest investigation, and management methods. Chinese authors who need to register their guidelines can do this on the International Practice Guideline Registration Platform (http://www.guidelines-registry.cn/).

4.4.2 Regarding proposal writing on the clinical practice guidelines for organic integration of TCM and WM, the following points should be considered

Does the guideline explicitly use integrated TCM and WM models of diagnosis and treatment? (For example, "combination of WM diseases and TCM syndrome differentiation", "combination of WM and TCM at different stages of disease", "syndrome differentiation and targeted therapy", "multidisciplinary diagnosis and treatment"). See **Appendix C** for details.

4.5 Formation of the guideline development team

The guidelines' development team should generally be composed of members with expertise in relevant clinical specialties (including TCM, WM, and integrated TCM and WM), evidence-based medicine, health economics, epidemiology, literature, statistics, and other professional skills. In additional relevant people from this and other fields should be included according to the appropriate content of the guideline, including inviting representatives from hospitals at different levels and patient representatives as members. Potential conflicts of interest must be considered before identifying members of the guideline development group. All panel members are required to conduct a conflict-of-interest survey and declare their interests (see **Appendix D** for details) and the results of all members' declaration-of-interest surveys will be published with the final guideline. In principle, relevant persons with significant conflicts of interest cannot participate in meetings related to the development of recommendations.

4.6 Synthesis and evaluation of evidence

4.6.1 General principles or methods

Generally, the types of evidence collected include guidelines, systematic reviews, meta-analyses, randomized controlled trials, observational studies, qualitative studies, professional consensus, expert opinion, case studies and economics studies.

According to the research methods used, types of evidence can be classified as primary and secondary research. In general, if a high-quality systematic review/meta-analysis is retrieved that matches the clinical question of the guidelines and has been published in the last 2 years, it can be used directly to save time and cost. However,

attention should be taken to ascertain whether any relevant literature has been published since the date of this systematic review search. If it is determined that there are no other relevant and high-quality systematic reviews/meta-analyses, then a systematic search of the original studies can be conducted. The literature for a particular study design should be selected based on the type of clinical question. In special cases, such as where the evidence has not been published, where there is no direct evidence, for rare diseases, for new diseases, etc., it may be difficult to retrieve appropriate research evidence. At this point, expert evidence may be the only or primary source of evidence.

The guidelines' formulation team needs to clearly define the inclusion and exclusion criteria for the literature, and rigorously apply the evaluation criteria of evidence-based medicine to scientifically evaluate the relevant literature. For example, quality evaluation of systematic reviews using the AMSTAR evaluation tool, quality evaluation of various types of original studies such as RCTs using the Cochrane Risk of Bias Assessment Tool, or the JBI principles of evaluation designed for specific studies.

The evidence evaluation should have resulted in an outcome summary table or evidence summary table (see **Appendix E**), which contains the PICO problems, the number of studies, the number of patients, outcome indicators and significance, a summary of the results for each outcome (including relative and absolute effect sizes), the quality of the evidence evaluation and the results of the evidence grading. This information provides a concise summary of the evidence for decision-makers to proceed to the generation of recommendations.

4.6.2 In response to the evidence search for clinical practice guidelines for organic integration of TCM and WM, the following questions should be considered

Has there been a complete retrieval of evidence sources for TCM, WM and integrated TCM and WM (such as consulting experts in the TCM field, searching the featured TCM database)?

Has there been a collection of characteristic evidence of TCM, such as TCM ancient books, expert experience?

Has there been a collection of evidence of patients' preference, willingness, and

values for accepting TCM or WM treatments?

Besides the above considerations, has there been a complete retrieval of information on the "body of evidence" for a specific clinical question? First, the "body of evidence" can be derived from a synthesis of evidence from the best research design, or it can be composed of evidence from multiple research methods and multiple sources. For example, when real world evidence exists, ancient documentary evidence and expert opinion evidence can be corroborated with real world evidence to form a "body of evidence". Again, for example, the "body of evidence" from randomized controlled trials provides evidence of intervention effectiveness, while studies such as observational studies or the registration and recording platforms of adverse reactions can provide research evidence of intervention implementation and safety, and the two types of evidence together support the designation of scientific and rational decision-making.

4.6.3 Considerations for evidence retrieval in the development of clinical practice guidelines for organic integration of TCM and WM

In the development of Guidelines for Organic Integration of TCM and WM, the evaluation of evidence sources needs to refer to the concept of "triple integration", which includes clinical research evidence, ancient literature and expert experience of TCM: none of the three can be ignored. However, a lot of work is still needed to deal with the definition of TCM expert opinions, because of the existence of overlap between the categorization of some expert opinions and the sources of ancient documents. In this standard, the expert opinions are those of contemporary medical practitioners, while the ancient literature refers to medical literature before 1911.

Ancient TCM literature and the experience of modern TCM experts should also be validated and evaluated by clinical studies (including traditional clinical studies or clinical studies based on real world data), so that they can enter the aggregation and evaluation of secondary studies and thus become a source of evidence for clinical practice guidelines. That is: the classical evidence of TCM is validated through the corroboration of ancient and modern evidence (**Fig. S3**).

If the expert opinion of traditional Chinese medicine must be used as the evidence

source, the evidence can be collected in the same way as the case report or case series study. For example, because there is very little research evidence that can account for the effects of the intervention, the experts can develop the overall number of their patients and estimate the effectiveness of the intervention, and then use structured forms to collect and analyze the above information and submit it to the guidelines' development team as a summary of the survey results.

The formulation of guidelines for Organic Integration of TCM and WM is often faced with difficulties because of insufficient evidence, especially the lack of high-quality studies on the comparison of integrated TCM and WM and non-integrated TCM and WM. At this point, the guidelines' development group needs to gather expert evidence that includes empirical feedback from long-term practice provided by people who are knowledgeable or technically skilled in a particular field, on the clinical issues that urgently need to be addressed. Unlike expert opinions, being collected systematically, expert evidence needs to provide facts to support the conclusion. And before the meeting forming the recommendations, expert evidence is provided to the consensus group members. If the evidence is properly summarized, expert evidence can be collected and evaluated in the same manner as a case report or a series of case studies.

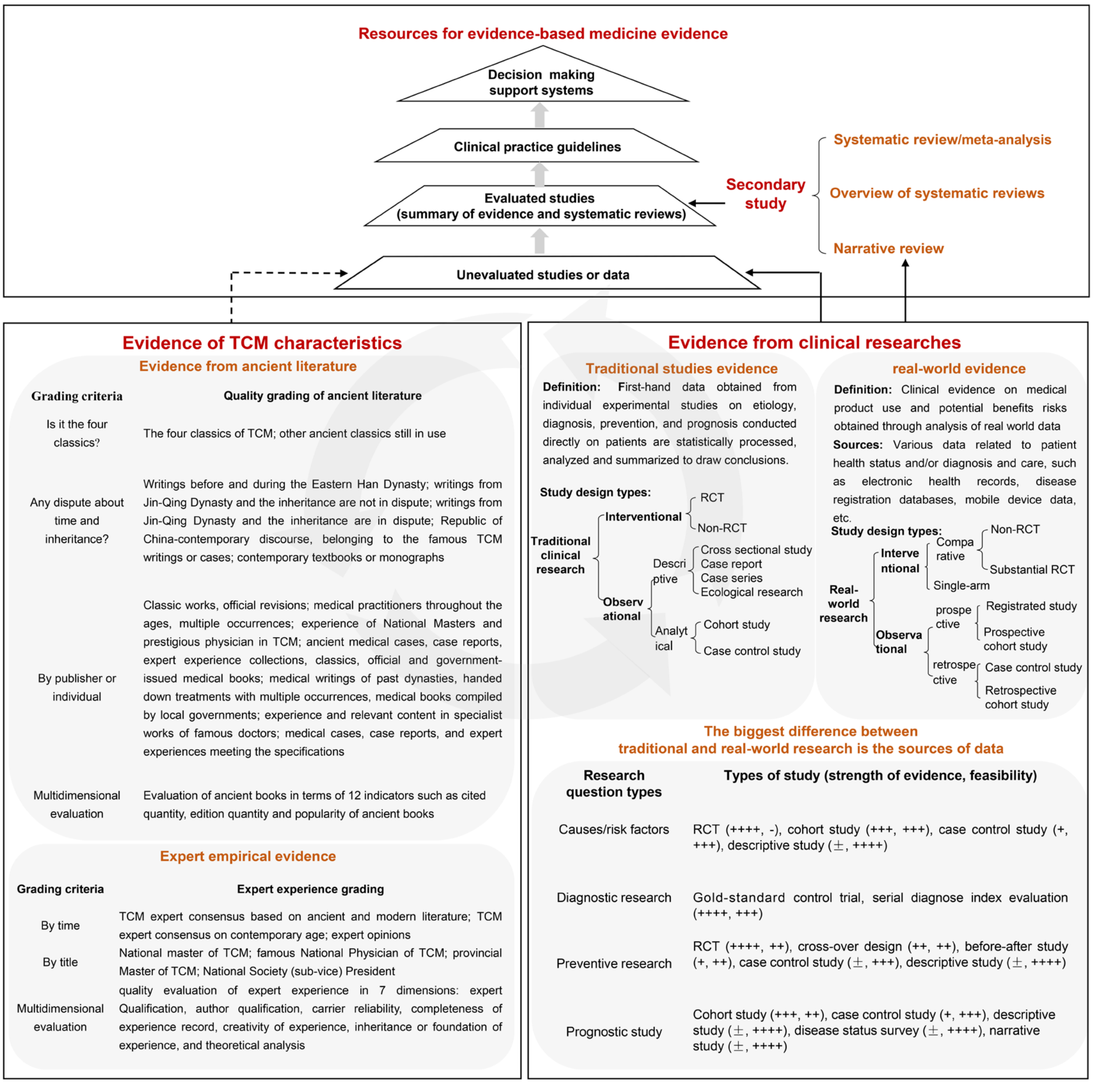

Fig. S3 Source levels of evidence developed and schematic diagram of the structure of clinical practice guidelines for organic integration of TCM and WM.

4.7 Quality of evidence and grading of recommendations

4.7.1 General principles or methods

The most commonly used international standard for grading the quality of evidence is GRADE. Through taking a comprehensive consideration of risk of bias, publication bias, inconsistency, indirectness, imprecision (random error), effect sizes, dose-response relationships, and confounding factors for included studies of systematic reviews, the GRADE evidence system classifies the effect indicators of systematic reviews as the "body of evidence" for quality assessment [6]. In addition, Chen et al [7]. have developed a revised version of the “Delphi” literature basis and grading of

recommendations (Chinese medicine), a grading system for recommendations, and the quality of evidence commonly used in guidelines/expert consensus for guidelines for TCM/WM, incorporating ancient and modern expert consensus and ancient literature into the quality of evidence grading and grading of recommendations. Chen et al [8]. have also developed the reference proposal for grading clinical research evidence and recommendations based on "body of evidence", incorporating expert opinions, experiences, and historical records, forming a grading standard for the quality of evidence based on body of evidence, so formulating a new grading standard for recommendations based on the GRADE standard.

4.7.2 In view of the evaluation of the quality of evidence and the classification of recommendations in the clinical practice guidelines for organic integration of TCM and WM, the following questions should be considered:

Is it necessary to classify the quality of evidence with TCM characteristics such as TCM ancient books and expert experience? Or is there a custom classification standard of the quality of evidence and recommendations for the Guidelines for Organic Integration of TCM and WM?

How should an appropriate grading system for the quality of evidence and recommendations be chosen in order to achieve a reasonable classification of included TCM and WM literature? In this process, is it necessary to make appropriate adjustments to the existing grading system of the quality of evidence and recommendations (especially the grading system based only on the modern medical system)? Has the adjustment method passed the consensus of the guide development group?

4.7.3 Points for attention in the formulation of clinical practice guidelines for organic integration of TCM and WM

It is reasonable and feasible to use the GRADE system to classify modern research evidence in the field of TCM, but there are still some challenges in the use of GRADE in guidelines for TCM / integrated TCM and WM. For example, evidence from ancient literature and expert experience is an important feature of TCM. When the evidence of modern medicine is insufficient and the evidence of ancient literature or expert

experience is sufficient, it will be difficult for guidelines developers to choose a rating if they directly choose to use GRADE system, and more exploration and thinking are needed in the field of TCM.

Acknowledging that the classical medical records of ancient books and the experience of famous experts are important supplements to the clinical evidence system of TCM, the existing guidelines of TCM / integrated TCM and WM often adjust and modify the GRADE system according to the specific evidence problems of TCM under the concept of the GRADE method, but this must be carried out based on the full consensus of the guideline-making methodologists and the formulation team.

The mixed use of multiple quality-of-evidence or recommended grading standards is not advocated, mainly because of the systematic nature and integrity of the grading standards in the process of development and formulation. If a complete grading standard is separated and/or combined with another standard, it is bound to break the internal logic of the original standard. For example, GRADE classification is based on different initial evidence levels of different research types, and then considers its promotion or downgrading. For different clinical problems, it takes into account the intensity, feasibility, advantages and disadvantages of the study design, especially the internal bias, as well as the cost and feasibility of transforming evidence into decision-making, which are not fully reflected in other classification standards.

4.8 Generation of recommendations

4.8.1 General principles and methods

Factors to be considered in the formation of recommendations: in addition to the quality of evidence, acceptability, feasibility, fairness, resource utilization, patients' values and preferences, and the balance of advantages and disadvantages are also factors that often need to be considered in the formulation of recommendations. In the actual operation, the above factors can be incorporated into the recommendation to form a framework for experts to judge item by item in the consensus meeting. In the process of formulating recommendations, the guidelines' working group should clarify and accurately define the factors to be considered in the formulation of recommendations, and quantify the

criteria to be considered as far as possible. It is suggested that the scientific and transparent formulation of recommendations should be ensured with the help of pre-designed content frameworks or auxiliary tools. As the formation of recommendations is affected by many factors, such as evidence, cost, and patient willingness, more and more evidence and recommendation classification standards no longer emphasize the one-to-one correspondence between recommendation intensity and evidence level, that is, high-level evidence may be weakly recommended and low-level evidence may also be strongly recommended. However, if the above situation occurs, it is recommended that the guidelines' development team should give a clear explanation.

The consensus method for the formation of recommendations: The consensus method is to collect a variety of suggestions or opinions from multiple individuals through a specific method to form a consistent conclusion or point of view. According to whether the formal consensus procedure or process is adopted, it can be divided into informal consensus method and formal consensus method. The informal consensus method is that participants fully express their own views, discuss them freely, and finally reach a consensus on recommendations. However, this lacks methods to reach a consensus, and individuals may not be able to fully express their true views because they are not familiar with the issue and feel pressure when they disagree with other people's views. In comparison, the formal consensus method adopts a structured process, clearly reaches a consensus, reduces the possible bias, and the results are more authoritative, reasonable and credible.

4.8.2 In view of the method for forming the recommendations for the clinical practice guidelines for organic integration of TTCM and WM, the following question should be considered:

When forming the recommendations for the organic integration of TCM and WM, are clinical experts from TCM, WM and the integration of TCM and WM invited to agree on the recommendations?

Does the generated recommendation explain the relationship between TCM treatment, WM treatment and integrated TCM and WM treatment? See Appendix F (recommendation Fusion example) for details.

4.8.3 Points for attention in clinical practice guidelines for organic integration of TCM and WM

TCM and WM have their own relatively complete diagnosis and treatment systems. TCM has TCM decoction, proprietary Chinese medicine, TCM injection, external treatment methods including acupuncture, cupping, moxibustion, Tuina, traditional skills of TCM and many other treatment methods. WM also has many treatment methods, such as Western medications, surgery, interventional therapy, physiotherapy, chemotherapy, and psychotherapy. When forming a recommendation, attention should be paid to the following:

a) In the analysis of the results of the evidence, for the sake of transparency in the formulation of the guidelines, attention needs to be paid to minimizing differences in importance of the outcome to assist in determining whether a recommendation is given, such as a reduction of at least 8 per 1000 people over five years and 10 per 1000 of non-fatal strokes.

b) In the choice of a TCM intervention or a WM intervention, the values and preferences of patients should be obtained by combining the theme of the guidelines and the use and cost of the intervention, so as to fully assess the absolute benefits and hazards of different interventions as well as the potential treatment burden.

c) According to the national conditions in our country, the guidelines for the organic integration of TCM and WM should absorb the theories of TCM and WM, highlight the advantages of the organic integration of TCM and WM, and give patients an individualized, accurate and humanized multi-mode diagnosis and treatment plan for integrated TCM and WM.

d) When formulating the guidelines, we should refine the best combination of "disease differentiation" in WM and "syndrome differentiation" in TCM, supplement the deficiency of "disease without syndrome" in WM, and make up for the embarrassment of "syndrome without disease and indefinite type" in TCM, so as to show the value of the combination of TCM and WM. It is necessary to refine the diagnosis and treatment modes of integrated TCM and WM, and form an accurate treatment method under the combination of syndrome differentiation, stage

differentiation and disease differentiation.

e) Under the guidance of the general principle of "cure the symptoms when it's urgent and cure the root causes when it's slow", we should promote the combination of dispelling pathogens and strengthening vital energy treatment, short-term cure and long-term regulation, and give full play to the complementary advantages of TCM and WM at different stages of treating the disease.

f) It is recommended that targeted recommendations are formulated according to different periods, different degrees of illness and different syndromes.

g) When the recommendations of TCM and WM are given at the same time, the order of treatments and priority should be clearly specified, for example, at which stage of the disease is the organic integration of TCM and WM better than simple TCM or simple WM? How should they be combined? Which is the main one? What are the potential interactions?

4.9 Writing of recommendations

4.9.1 General principles and methods

Recommendation intensity is the degree to which guidelines' developers comprehensively consider the quality, feasibility and acceptability of evidence, patients' values and wishes and other factors to understand the advantages and disadvantages of the implementation of recommendations. This must normally be described using technical terms and symbols and generally appears after the text of each recommendation. The text of the recommendation must be clear and feasible. The use of clear text language to express the recommended content is important and where necessary should include an explanation of the relevant technical terms or content. Each recommendation should specify its recommendation intensity, and the description of recommendation intensity generally refers to the use of clear language or symbols after the recommendation sentence to distinguish the different levels of recommendation intensity. In addition to marking the level of recommendations, reflecting the level of recommendations is proposed by describing them in standard terms, such as "suggestions" and "offer" to reflect strong recommendations and "considerations" to

reflect weak recommendations. All these ensure that recommendations are clearly expressed and consistent in the full text of the guide.

Recommendations should not appear in large sections of the guidelines as far as possible, but should be summarized separately, or presented in bold, underlined form, or in the form of a table, which is clear, concise and easy to understand, and provides a direct reference for users.

4.9.2 Points for attention in clinical practice guidelines for organic integration of TCM and WM

When there is no evidence of the effect of the organic integration of a TCM and WM, it may not be possible to make recommendations based on the evidence, in which case, a statement can be made such as "because there is no evidence of the effectiveness (or harm) of intervention X, it is not possible to make a clear recommendation" or "unable to make recommendations due to lack of evidence". Sometimes, although there is no good evidence, the guideline from professional institutions is still needed (for example, clinical research shows that this problem is an urgent clinical problem), and at this time the guideline-making team should also make recommendations accordingly, which can be marked "based on the recommendations from a consensus of experts". For example, in the absence of strict research evidence, the evaluation of ancient books, medical records of TCM and WM experts can also become the basis for the formulation of recommendations, but their retrieval, selection, synthesis and evaluation methods should be determined in the guidelines plan and consistent with the quality of evidence of this guidelines and the classification method of recommendation opinions, and marked with a statement such as "consensual recommendation" or "expert consensus" after the corresponding recommendation opinion.

4.10 External examination and evaluation

After the formation of consensus recommendations, the guidelines development group should invite experts in relevant fields outside the formulation group, experts in the field of integrated TCM and WM, and other stakeholders as external assessors. In order to ensure the quality of the external audit, the external auditors must also submit the

relevant declaration of conflict of interest. The external auditors can make comments through a variety of channels; the most common way of achieving this is to fill in the form. The content of the table generally revolves around the accuracy and clarity of the recommendations, the rationality of the guidelines' formulation method, especially the completeness and correctness of literature retrieval and evaluation, and the transparency of the guidelines' formulation process.

There are two main aspects to external audit: opinion solicitation and peer expert review. The purpose of soliciting opinions is to introduce the preliminary conclusions and relevant recommendations of the guidelines to different groups in society through various channels after writing the draft guidelines. Expert opinions are extensively solicited to ensure the applicability and extensibility of the guidelines. The peer review is an invitation to peer experts to review the guidelines from a professional perspective after the guidelines have been revised and refined by the guidelines development team based on the comments collected.

4.11 Report of clinical guidelines

4.11.1 General principles and methods

The content of the report of guidelines generally includes abstract, introduction, main clinical problems, evidence, recommendations, reasons for recommendations, attached references, acknowledgments, list of personnel. The WHO handbook for guideline development proposes [9-10] that all guidelines should include executive summary, main body, and appendices, the executive summary should contain the main recommendations of the guidelines, and the main body should contain the table of contents, introduction, methods, recommendations, and conclusions. In addition, the systematic review and grading of results can be presented in the appendices. After the guidelines have been developed, they should be checked to ensure a full and complete report according to the Reporting Items for Practice Guidelines in Healthcare (RIGHT) inventory and checklist for the reporting of guidelines of TCM to fully consider reporting details from recommendation of TCM treatment. After the recommendations have been written, it is recommended that the guidelines' development team produce a

flowchart of the implementation of the recommendations, pictures and videos or digital intelligent solutions which facilitate the implementation of the recommendations to be published together with the text of the guidelines or give download links in the guidelines text.

4.11.2 In response to the report of clinical practice guidelines for organic integration of TCM and WM, the following questions must be considered

Does the title of the guidelines clearly state "guidelines for organic integration of TCM and WM?"

Has a recommendation implementation pathway map or other visual form been developed to present the recommendation, which reflects the full organic integration of TCM and WM recommendations?

Is the report of clinical practice guidelines of organic integration for TCM and WM based on the pre-set model of integration of TCM and WM?

4.12 Promotion and implementation of guidelines

The guidelines' development organization has the responsibility and obligation to promote the dissemination and promotion of the guidelines.

In the process of guidelines implementation, obstacles may be encountered at multiple levels, and the process of guidelines promotion should take note of these obstacles and give reasonable suggestions to deal with them. Meanwhile, experts in the field can develop matched implementation strategies for the guidelines.

4.13 Updating of guidelines

Guidelines should be updated 2 - 3 years after publication. The guidelines panel should undertake long-term follow-up on the evidence related to guidelines recommendations after the guidelines are published.

The international trend in guidelines development emphasizes the dynamic development of systematic reviews, i.e., dynamic development through living systematic review. The guidelines update process is initiated when new relevant evidence emerges from new guidelines publication and the change in evidence has an impact on the strength or direction of this guideline's recommendations, at this point,

the update may not be limited to 2 - 3 years after guidelines publication. Guidelines updates should be published in a timely manner through the guidelines update organization's website and journals, and the updated content should be prominently and clearly marked.

The above guideline development methods are not sufficient to meet the whole process and methodological details of a guideline development. The guideline development team needs to integrate the above methodological points into the guideline development process in a sequential manner (see Appendix G). Guidelines' developers can still refer to the basic methodology of other published guidelines (especially in the field of WM) to understand many details of the guidelines' development process, such as the contributors and their role in guidelines development, literature retrieval and systematic review of the guidelines' development team.

Appendix A

Proposed issues for clinical practice guidelines for organic integration of Traditional Chinese and Western medicine

Overview

This appendix gives examples of clinical problems that have been raised during the development of clinical practice guidelines for organic integration of traditional Chinese and Western medicine in the categories of treatment/intervention, prognosis, injury, or etiology.

Clinical problems related to "diagnosis" are not shown in the examples, mainly because the diagnostic questions proposed by evidence-based approach are usually a study of the accuracy of the diagnostic methods to be evaluated in comparison with the gold standard, while TCM and WM belong to two completely different diagnostic systems, the diagnostic problems related to the integration of TCM and WM are often to judge the compatibility rate of syndrome types in TCM with Western biochemical or imaging examination. Although the research results of such literature can prove the material basis of syndrome types in TCM in modern medical research, they are of limited significance to the clinical diagnosis and treatment practice concerned by the guidelines, so such diagnostic problems are not included in the content of this example.

In practice, there may be background clinical problems in the formulation of clinical practice guidelines for organic integration of TCM and WM, such as "What are the syndrome types of muscle and bone pain/diagnostic criteria in TCM", which need to be put forward according to the actual situation.

A.1 Therapeutic/interventional clinical problems

Examples of clinical problems posed in the treatment/intervention category are shown in **Table.A1**.

Table.A1 Treatment/intervention

Category	Clinical problems

**Treatment/Intervention**	In men with premature ejaculation, did oral paroxetine combined with Shugan Yiyang capsules prolong intravaginal ejaculation latency time (IELT) after 8 weeks off the drug compared with oral paroxetine alone? Did the Chinese index of sexual function for premature ejaculation scale (CIPE) score improve?<br>**Population:** Men with premature ejaculation (WM disease name)<br>**Intervention:** Oral paroxetine combined with Shugan Yiyang capsules<br>**Comparison:** Oral paroxetine alone<br>**Outcome:** 1. The IELT after 8 weeks off the drug; 2. The CIPE score
	In elderly patients with gastrointestinal dysfunction due to sepsis, which method is more effective in improving patients' syndrome of spleen-stomach Qi deficiency compared with conventional WM treatment by combining this with Xiang-sha six jun-zi decoction?<br>**Population:** Elderly patients with gastrointestinal dysfunction due to sepsis (WM disease name)<br>**Intervention:** Xiang-sha six jun-zi decoction combined with conventional WM treatment<br>**Comparison:** Conventional WM treatment<br>**Outcome:** 1. Score of syndrome of spleen-stomach Qi deficiency scale; 2. Scale of gastrointestinal dysfunction score

Table.A1 Treatment/intervention (Continued)

Category	Clinical problems
**Treatment/Intervention**	In patients with chronic renal failure, which is more effective in improving edema combining oral keto acid tablets with reserved enemas in TCM (30 g calcined longgu, 30 g calcined oyster, 30 g dandelion) compared with oral keto acid tablets alone?<br>**Population:** Patients with chronic renal failure (WM disease name)<br>**Intervention:** Oral keto acid tablets combined with reserved enemas in TCM treatment<br>**Comparison:** Oral keto acid tablets<br>**Outcome:** Ankle circumference difference
	In patients with ulcerative colitis, oral mesalazine combined with Chinese herbs (basic formula of Chinese herbs: Astragalus 30 g,

	cinnamon 10 g, ginger 10 g, patrinia 20 g, coptis 10 g, atractylodis macrocephalae koidz 15 g, atractylodes 15 g, natural indigo15 g, bitter ginseng 15 g, salvia miltiorrhiza 15 g, roasted licorice 10 g; when there is obvious abscess and blood in stool add anemoside 15 g, cortex fraxini 12 g; when there is urgency and posterior heaviness, add bupleurum 12 g, cimicifugae rhizoma 69 g) Compared with oral mesalazine alone, which method is more effective in improving patient's diarrhea?<br>**Population:** Patients with ulcerative colitis (WM disease name)<br>**Intervention:** Oral mesalazine combined with TCM<br>**Comparison:** Oral mesalazine<br>**Outcome:** Frequency of diarrhea
	In patients with diabetic nephropathy, which regimen is more effective in improving renal function (SCr and BUN) and 24 h urine protein quantification in patients treated with hemiprostone combined with prostaglandin compared to hemiprostone alone or prostaglandin alone?<br>**Population:** Patients with diabetic nephropathy (WM disease name)<br>**Intervention:** Hemiprostone combined with prostaglandin<br>**Comparison:** 1. Hemiprostone; 2. Prostaglandin<br>**Outcome:** 1. Renal function (SCr and BUN); 2. 24 h urine protein quantification
	In patients with cerebral infarction, which is more effective in improving patients' neurological deficits and daily living ability, the combination of aspirin and Buyang huanwu decoction compared with aspirin alone?<br>**Population:** Patients with cerebral infarction (WM disease name)<br>**Intervention:** Aspirin in combination with Buyang huanwu decoction<br>**Comparison:** Aspirin<br>**Outcome:** 1. The national institute of health stroke scale; 2. The activity

	of daily living scale
	In patients with functional dyspepsia, which method is more effective in treating patients' gastric motility and restoring normal levels of gastrointestinal hormones, using Tuina massarge versus oral cisapride?<br>**Population:** Patients with functional dyspepsia (WM disease name)<br>**Intervention:** Tuina<br>**Comparison:** Oral cisapride<br>**Outcome:** 1. Gastric motility (The number of migrating compound motor waves (MMC) and postprandial sinus contraction amplitude P, contraction frequency F); 2. Serum gastrointestinal hormone levels (Gastrin, growth inhibitor, motilin)

Table.A1 Treatment/intervention (continued)

Category	Clinical problems
**Treatment/Intervention**	In the treatment of patients with eczema of the yin deficiency and wet stagnation, which method is more effective in improving the area and severity index of eczema and the degree of itching? The use of oral Chinese herbs (rhizoma picrorhizae, forsythia, gypsum, poria, atractylodes, danpi, adenophorae radix, oyster, adenophorae radix, adenophorae radix, red bean, schizonepeta, radix paeoniae, bitter ginseng, astragalus) compared with oral levocetirizine hydrochloride combined with topical compound flumethasone?<br>**Population:** patients with eczema of the yin deficiency and wet stagnation syndrome (TCM syndrome type and WM disease name)<br>**Intervention:** Oral Chinese herbs<br>**Comparison:** Oral levocetirizine hydrochloride combined with topical compound flumethasone<br>**Outcome:** 1. The area of eczema; 2. The severity index of eczema; 3. The degree of itching
	In patients with shift work sleep disorders, which method is more effective in

	improving sleep quality, head point Tuina therapy combined with oral eszopiclone versus oral eszopiclone tablets?<br>**Population:** Patients with shift work sleep disorders (WM disease name)<br>**Intervention:** Head point Tuina therapy combined with oral eszopiclone<br>**Comparison:** Oral eszopiclone tablets<br>**Outcome:** The Pittsburgh Sleep Quality Scale

A.2 Prognostic clinical problems

Examples of clinical problems posed in the prognostic category are shown in **Table.A2**.

Table.A2 Prognosis

Category	Clinical problems
	For HIV patients, what factors reduce patients' mortality?<br>**Population:** HIV patients (WM disease name)<br>**Exposure:** 1. Combined TCM treatment group on the basis of standardized WM treatment 2. Acute phase<br>**Comparison:** 1. Standardized WM treatment 2. Non-acute phase (asymptomatic, pre-AIDS and AIDS phase)<br>**Outcome:** Mortality rate of AIDS patients
**Prognosis**	For elderly patients with gastric cancer, what factors can affect survival?<br>**Population:** Elderly patients with gastric cancer (WM disease name)<br>**Exposure:** 1. Tumor node metastasis classification; 2. Male; 3. Radical surgery<br>**Comparison:** 1. Tumor node metastasis classification (compare with each other); 2. Female 3. Non-radical surgery<br>**Outcome:** 1. Overall survival; 2. Disease free survival
	In patients with post-operative PTCA for coronary artery disease, whether different syndromes in TCM will affect the incidence of restenosis?<br>**Population:** Patients with post-operative PTCA for coronary artery disease (WM disease name)<br>**Exposure:** 1. Phlegm syndrome; 2. Qi stagnation syndrome; 3. Blood stasis syndrome; 4. Cold condensation syndrome<br>**Comparison:** 1. Non-phlegm syndrome; 2. Non-qi stagnation syndrome; 3. Non-blood stasis syndrome; 4. Non-cold condensation syndrome

	**Outcome:** The incidence of restenosis
	What factors affect the prognosis for adult acute lymphoblastic leukemia patients treated with a combination of TCM and WM?<br>**Population:** Adult acute lymphoblastic leukemia patients (WM disease name)<br>**Exposure:** 1. Qi and blood deficiency syndrome; 2. Heat toxicity into the blood type syndrome; 3. Positive Ph chromosome<br>**Comparison:** 1. Non-qi and blood deficiency syndrome; 2. Non-heat toxicity into the blood type syndrome; 3. Non-positive Ph chromosome<br>**Outcome:** Survival rate

HIV human immunodeficiency virus, *AIDS a*cquired immune deficiency syndrome

A.3 Examples of clinical problems posed in the injury/etiology category are shown in Table.A3

Examples of clinical problems posed in the injury/etiology category are shown in **Table.A3.**

Table.A3 Injury or etiology

Category	Clinical problems
**Injury/Etiology**	Whether phlegm, qi stagnation, blood stasis, and heat accumulation will increase the risk of anxiety in patients with slow coronary artery flow?<br>**Population:** Patients with slow coronary artery flow (WM disease name)<br>**Exposure:** 1. Phlegm; 2. Qi stagnation; 3. Blood stasis; 4. Heat embodiment<br>**Comparison:** 1. Non-phlegm 2. Non-qi stagnation 3. Non-blood stasis 4. Non-heat embodiment<br>**Outcome:** Hamilton Anxiety Scale (HAMA)
	Whether HP infection increases the risk of spleen and stomach disorder functional dyspepsia in patients with functional dyspepsia?<br>**Population:** Patients with functional dyspepsia (WM disease name)<br>**Exposure:** HP infection<br>**Comparison:** Non-infected with HP<br>**Outcome:** Incidence rate of functional dyspepsia with spleen and stomach disharmony
	Does evidence of kidney deficiency increase a woman's risk of hypomenorrhea?

	**Population:** Female<br><br>**Exposure:** Kidney deficiency syndrome<br><br>**Comparison:** Non-kidney deficiency syndrome<br><br>**Outcome:** Incidence rate of hypomenorrhea
	Does Yin and Yang deficiency increase the risk of retinal vasculopathy in patients with primary hypertension compared to hyperactivity of liver fire?<br><br>**Population:** Patients with hypertension (WM disease name)<br><br>**Exposure:** Yin and Yang deficiency<br><br>**Comparison:** Hyperactivity of liver fire<br><br>**Outcome:** Incidence rate of retinal vasculopathy

Appendix B

Problems with clinical practice guidelines for organic integration of traditional Chinese and Western medicine and the importance of outcome indicators

Judgment of the importance of clinical problems

B.1 Importance of judgment of clinical problems

Table.B1 shows questionnaires about importance of judgment of clinical problems.

Table.B1 Significance scoring scale for clinical problems

Please make a judgment about the importance of the research question involved in this guideline, and type "√" on the corresponding score value (using the Likert 5-level scoring method: 5 = very important, 4 = important, 3 = general, 2 = not important, 1 = very unimportant).

Item content	Importance				
Question 1	5	4	3	2	1
Question 2	5	4	3	2	1
……	5	4	3	2	1
Please add any important issues that we may have missed, and grade them					
	5	4	3	2	1
Note:					

The problem importance score can also be scored using 1-7 points.

B.2 Importance of judgment of outcome indicators

Table. B2 shows the judgment assigned to the importance of clinical outcome indicators.

Table. B2 Significance scoring scale of outcome indicators

Please judge the importance of the following outcome indicators and fill in the corresponding box that you think is the most appropriate score (Score on a 9-point scale by importance level, 7 to 9 is "key outcome", 4 to 6 "important outcome", and 1 to 3 "general outcome"). If you need to supplement some outcome indicators for the

corresponding clinical problems, please fill in the corresponding blank box and give the corresponding score value (classification A and classification B, such as efficacy outcome indicators and safety outcome indicators).

Classify A		Classify B		……	
Indicator A1		Indicator B1		……	
Indicator A2		Indicator B2		……	
……		……		……	
Please add any important outcome indicators that we may have missed, and score them		Please add any important outcome indicators that we may have missed and score them		Please add any important outcome indicators that we may have missed and score them	

Appendix C

Organic integration of traditional Chinese and Western medicine model

C.1 Combination of WM diseases and TCM syndrome types model

The mode of combination of WM diseases and TCM syndrome types is a way to combine the "syndrome differentiation" of TCM with the "symptom differentiation" of modern medicine. This model comes from the summary of the practice of Integrated TCM and WM for more than half a century, and is a relatively successful clinical research model for Integrated TCM and WM, reflecting the combination of the overall thinking, imagery thinking, dialectical thinking of TCM and modern medical science materialism, and reflecting the combination of the integrated macroscale of TCM and the local micro of modern medicine [11].

Disease differentiation is the characteristic of WM in diagnosis, focusing on the whole process of pathological changes of the disease, emphasizing the internal law of physiological and pathological changes [12]. Syndrome differentiation is the characteristic of TCM in diagnosis, which focuses on the overall understanding of the disease state at a certain stage, and focuses on the different state of each patient's body and its different environment. WM is diagnosed through the physical examination by observation, palpation, percussion and auscultation to collect data about the disease, combining modern examinations, pathology, imaging, and other assessments to determine the diagnosis. This method of diagnosis and treatment is both intuitive and microscopic. Syndrome differentiation in TCM refers to treatment based on disease data collected by observation, smell, questioning, and pulse-taking, analyzing the cause and pathogenesis, identifying the nature and location of the disease and the process of the rise and fall of pathogenic factors. This method of diagnosis and treatment is macroscopic and integrated. The mode of combination of WM diseases and TCM syndrome types is the basic clinical and scientific research method used in Integrated TCM and WM. The characteristic of this mode is often reflected in the following six aspects [13]:

a) Combined disease and syndrome, dual diagnosis. Using the diagnostic means and methods of modern medicine, diagnose clearly what the disease is (including

pathological diagnosis), and identify what the syndrome is according to the TCM syndrome types differentiation method.

b) Disease differentiation is the main method, syndrome differentiation is the supplementary method. In clinical treatment, it is necessary to comprehensively consider both the characteristics of modern medical disease diagnosis and the characteristics of TCM syndrome diagnosis, prescribe drugs for the key pathological problems of the disease, and at the same time supplement this according to the results of TCM syndrome differentiation and treatment with drugs for the syndrome.

c) Syndrome differentiation is the main method, disease differentiation is the supplementary method. According to the patient's information of four diagnoses, to identify the syndrome diagnosis of disease and the corresponding prescription. On this basis, appropriate consideration is given to the use of disease-specific drugs in the light of the understanding of disease pathology in modern medicine and the results of pharmacological research.

d) Microcosmic syndrome differentiation. When a patient without any clinical symptoms, only has abnormal physical and chemical indicators, a comprehensive analysis can be made according to the patient's past medical history, physical tendency, physical and chemical indicators and the research results of modern TCM micro syndrome differentiation.

e) Syndrome without disease, disease without syndrome. "Syndrome without disease" is directly selecting the prescription herbs for the syndrome according to the results of TCM syndrome differentiation. "Disease without syndrome" is directly selecting prescription drugs for pathology according to the pathophysiological mechanism of the disease.

f) Combined TCM and WM, dual therapy. In view of the diseases diagnosed by WM, it is better to take Western drugs according to symptoms differentiation combined with syndrome differentiation in TCM than to use Chinese or Western medicine alone.

Disease differentiation is helpful to improve the accuracy of syndrome differentiation, and syndrome differentiation is helpful for the concretization of disease differentiation.

On the one hand, the content of the four diagnoses of TCM can be enriched and supplemented by the help of examination, pathology, imaging and other means. On the other hand, for some diseases with abnormal indicators without obvious clinical manifestations, the combination with WM can deal with the dilemma of unidentified syndromes in TCM. For some diseases that cannot be diagnosed positively by WM examination, the combination with TCM syndrome differentiation can make up for the lack of disease identification in WM.

C.2 Combination of WM and TCM at different stages of disease model

The mode of the combination of disease stage and syndrome is to differentiate the syndrome at different stages of the disease on the premise of making clear the diagnosis of the disease by WM, to combine the disease stage and syndrome vertically based on the combination of the disease and syndrome, and to differentiate and analyze the syndrome from dynamic multi-dimensional angles. Diseases can be staged according to the WM diagnosis, considering the disease process, the severity of the disease, and the test indicators [14].

To establish a modern diagnosis and treatment model of integrated TCM and WM based on "syndrome differentiation and treatment, combined with disease stage and syndrome", it is necessary to find the best combination point of "disease differentiation" and "syndrome differentiation" in WM and TCM, to supplement the deficiency of "disease without syndrome" in WM and make up for the embarrassment of "syndrome without disease or stages" in TCM, which shows the unique guiding value of Integrated TCM and WM. Refine the combination model of "macroscopic differentiation of syndromes + differentiation of stages + microscopic differentiation of diseases" to form an optimized treatment that combines syndrome differentiation, stage differentiation, and disease differentiation[15].

C.3 Mode of syndrome differentiation and targeted therapy

The mode of syndrome differentiation and targeted therapy involves combining the macro "state" and "syndrome" of TCM with the micro "symptom" and "target" to communicate the macro and micro diagnosis and treatment in integrated TCM and WM [16].

On the one hand, the idea of "syndrome differentiation and targeted therapy" requires "correction" of imbalances. From a macro perspective, when considering the state of cold and heat and the imbalance of Yin and Yang by diseases, the partial nature of drugs is used to restore the self-harmony of Yin and Yang in the human body and lead to recovery from disease. In view of diseases that have been unambiguously diagnosed in modern times, there needs to be a clear understanding of the physiological and pathological features of the disease identified with the help of modern medicine. The whole process of the disease needs to be examined according to TCM thinking, the characteristics of various stages of the disease development must be clarified, and the core pathogenesis for syndrome differentiation and treatment must be summarized. On the other hand, it emphasizes the clinical "targeting", that is, to improve the targeting and precision of clinical treatment. The targeting of clinical treatment has three aspects: targeting the disease itself, targeting the typical symptoms, and targeting the clinical physical and chemical indicators. The research results of modern pharmacology are used to improve the precision of TCM prescription drugs and accurately target specific diseases.

In addition to the above, there may also be other modes of organic integration of TCM and WM for its specific clinical practice themes, such as the mode of syndrome combination (symptoms in WM and syndrome in TCM). The guideline development team also needs to discuss this in-depth to fit it into clinical practice.

Appendix D

Conflict of interest statement

Overview

All experts on the guideline steering committee, the guideline consensus group, the guideline development working group and the guideline external review group must disclose all potential conflicts of interest (e.g. all interests that affect or may affect the objectivity and independence of the experts) using the form below.

On this conflict of interest statement, you must disclose any commercial, professional or other conflicts of interest related to the subject of this guideline, and any interests that may be affected by the results of this guideline.

D.1 Experts' basic information

Experts' basic information should contain the information shown in **Table. D1**.

Table. D1 Experts' basic information

Name		Gender		Professional field	
Institutions		Rank		Position	
Phone number			E-mail		
Correspondence address					

D.2 Financial conflict of interest statement

The financial conflict of interest statement should contain the information shown in **Table.D2**.

Table.D2 Financial conflict of interest statement

Within the past 36 months:	
I or my immediate family members have owned technology, shares, or stocks in companies, institutions, or organizations that have an interest in the guideline.	YES□ NO□
I or my immediate family members have had paid employment, consulting, or other relationship with companies, institutions, or organizations that have an interest in the guideline.	YES□ NO□

I or my immediate family members have received fees, equipment, services, etc. from companies, institutions, or organizations that have an interest in the guideline to support research.	YES□ NO□
I or my immediate family members have received more than 10,000 RMB of other fees or services (e.g., travel expenses, gifts, vocational training, etc.) from companies, institutions, or organizations that have an interest in the guideline.	YES□ NO□

Note 1: Companies, institutions or organizations that have an interest in the guideline refer to companies, institutions or organizations that develop, manufacture, sell, promote, and provide products or services that are related to medical devices, equipment, drugs, preparations, etc. involved in the recommendations of the guideline.
Note 2: Immediate family members refer to those who have direct blood relationship or marriage to the expert, including a spouse, parent, and children.

D.3 Non-financial conflict of interest statement

The non-financial conflict of interest statement should contain the information shown in Table.D3.

Table.D3 Non-financial conflict of interest statement

**Within the past 36 months:**	
I have published academic papers related to the contents of the guideline	YES☐ NO☐
I have applied for a technical patent related to the content of the guideline	YES☐ NO☐
I have religious, political, ideological and other personal beliefs related to the content of the guideline	YES☐ NO☐
I have personal relationships with authors, editors, etc. of academic papers involved in the guideline	YES☐ NO☐

In addition to the above, within the past 36 months, is there anything else that you need to declare? If yes, please fill in the box below.

YES☐ NO□

D.4 Declaration of interest publication of informed consent and authenticity

In order to ensure the authenticity and validity of the declaration, the conflict of interest declaration should be signed by the declarer.

Example:

**Informed about the conflict of interest statement:** I agree to make the above completed content public to other guideline development members, and agree that the statement of interest form will be published in the guideline.<br>**Statement:** I promise that what I have stated is true and complete. If the information I have stated above changes at any time, I will promptly inform the guideline secretary group and complete a new statement of interest form.<br><br>**Signed: ________________ Date: ________**

Appendix E

Evidence summary table and consensus voting table

Table. E1 Summary of evidence

Quality assessment		No of patients		Effect		Quality of evidence/Level of evidence	Importance of outcomes
No. of studies	Study design	Intervention group	Control group	Relative (95% CI)	Absolute		
Outcome 1							
Outcome 2							
Outcome 3							
Outcome 4							
Outcome 5							
Outcome 6							
Outcome 7							

Note 1: The maximum number of outcome measures was 7;

Note 2: Outcome measures were divided into three levels according to their importance: critical, important, and not important;

Note 3: If GRADE assessment is used, descriptions of downgrading or upgrading factor results may also be added to the table.

E.2 Consensus voting table is shown in Table. E2

Table. E2 Consensus Voting Table

Considerations for formulating recommendations	Direction of	Strength of

		recommendations	recommendations
Quality of evidence/Level of evidence		☐Strong for<br>☐Weak for<br>☐Against	☐Strong<br>☐Weak
Cost of intervention	☐Low<br>☐Medium<br>☐High		
Patients' wishes (non-economic factor, intervention is acceptable to the patient)	☐Accept<br>☐May not accept<br>☐May not be consistent<br>☐Unclear		
Feasibility of intervention	☐Low<br>☐Medium<br>☐High		
Pros and cons of judgment	☐Favorable outcome is much more likely than unfavorable outcome<br>☐Favorable outcomes may be more likely than unfavorable outcomes<br>☐The pros and cons are equal<br>☐The unfavorable outcomes may be more likely than favorable outcomes<br>☐The pros and cons are unclear		

Note 1: The factors influencing recommendations can be added or deleted according to the actual situation;

Note 2: The wording of the form needs to be changed according to the specific question, such as "intervention" may need to be replaced with a specific measure or a specific diagnostic protocol;

Note 3: The direction and strength of recommendations can be expressed in different forms, such as A/B/C classification, etc., and the options in the table can be modified

according to the actual situation.

Appendix F

Examples of relationship forms applied in recommendations for clinical practice guidelines for organic integration of traditional Chinese and Western medicine

Table. F1 shows examples of recommendations which can reflect the intervention relationship between traditional Chinese and Western medicine.

Fig. F1 shows recommendation flow chart and the relationships between Integrated TCM and WM by referring to part of the published guidelines.

Table. F1 Examples of recommendations

Relationships between integration and separation of TCM and WM	1. In order to improve the neurological function of patients with cerebral infarction, Chinese herbs for promoting blood circulation and removing blood stasis can be considered within 6 hours after the disease onset in combination with intravenous thrombolysis therapy after strict exclusion of contraindications. The determination of medication timing should be considered alongside the patients' bleeding risk and specific conditions. (2C)<br>2. Extrauterine pregnancy recipe I integrated with WM can be used in patients with a differential diagnosis of undamaged stage - fetal obstruction syndrome, blood β-hCG ≤ 2000 U/L, tubal pregnancy mass diameter ≤ 4 cm or damaged stage - positive deficiency and blood stasis syndrome, score ≤ 9 points. (2C)<br>3. In addition to routine WM treatment, TCM is recommended to reduce uric acid and delay the progression of renal damage in patients with gouty nephropathy. To improve renal function, oral TCM decoction or proprietary medicine should be used according to syndrome differentiation, and should be reasonably combined with external treatment such as TCM retention enema. (1B)<br>4. For patients with tubal pregnancy treated with WM, it is recommended for this to be combined with TCM, such as extrauterine pregnancy recipe I or II. (1B)<br>5. For the patients with small hepatocellular carcinoma, the combination of oral decoction and Chinese herbal anti-tumor injection can reduce the risk of recurrence to some extent. (2C)<br>6. For patients with HBV necrosis stage, the integration of immunosuppressive TCM and WM, such as the use of short-range hormones in the early stage of necrosis, combined with TCM for cooling blood, detoxifying and removing blood stasis as this can reduce the incidence of HBV ACLF and the degree of inflammation.<br>7. For patients with HBV plateau, using TCM for tonifying Qi and warming Yang to stimulate positive Qi, combined with immunopotentiator could reduce infection or improve the inflammatory intervention effect and reduce the proportion of patients going on to end-stage progression.<br>8. For patients with severe pancreatitis during the systematic infection stage, anti-infection, systemic support and combined treatment with TCM are recommended, and surgical treatment should be performed when there are clear signs of infection. The main treatment which combines TCM for cooling blood and detoxifying, promoting

	blood circulation and removing blood stasis with removing stasis by purgation, invigorating Qi and activating blood circulation is recommended by TCM. Minimally invasive surgery which can remove necrotic tissue is recommended when patients have symptoms of infection and necrosis.<br>9. For convalescent COPD patients with pulmonary function level 3 and 4, formulating traditional Chinese treatment protocols of syndrome differentiation based on WM treatment is recommended.
Recommended TCM interventions	1. Western medicine has no specific treatment for cytokine storm or dysfunctions of blood coagulation in the early disease stages, as well as in drug-resistant bacterial infection, endotoxemia and toxic megacolon in the plateau and terminal stage. Clinically, the intervention effect of TCM therapies including cooling blood, detoxifying, purgating fu-organ, Fuzheng, warming. Yang and Tongqiao, is explored. By actively sending medicine formula developed in TCM principle-methodology-formula-medication and using Western pathophysiology as the reference.<br>2. The combination of Huachansu injection and arsenic trioxide injection or their replacement for some chemotherapy drugs in TACE surgery could reduce adverse reactions to chemotherapy, improve patients' quality of life and to a certain extent further improve clinical efficacy compared with chemotherapy drugs alone. (2B)
Recommended WM interventions	1. For patients with hemorrhagic stroke, using surgical treatment is strongly recommended when: 1) there is temporal lobe hernia; 2) CT and MRI show that the midline structure has shifted more than 5mm; the ipsilateral lateral ventricle has been compressed and occluded more than 1/2; the ipsilateral cerebral cisterna and sulcus have obscured or disappeared; 3) intracranial pressure＞25 mmHg.
TCM interventions Not recommended	1. For cerebral infarction patients treated after 6 hours of onset, TCM for promoting blood circulation and removing blood stasis is not recommended in combination with intravenous thrombolysis therapy. (2C)<br>2. For cerebral infarction patients in the acute stage of internal hemorrhage, TCM for promoting blood circulation and removing blood stasis is not recommended in combination with routine WM treatment.<br>3. For patients with hypertensive cerebral hemorrhage, taking TCM for promoting blood circulation and removing blood stasis within 3 days of onset is not recommended.
WM interventions Not recommended	1. For PLC patients in the middle and advanced stages for whom surgery or TACE treatment is not an option, general symptomatic and supportive treatment with intravenous infusion of anti-tumor TCM could improve quality of life and clinical benefit rate. (2B)

β-hCG β-human chorionic gonadotrophin, *TCM* traditional Chinese medicine, *WM* western medicine

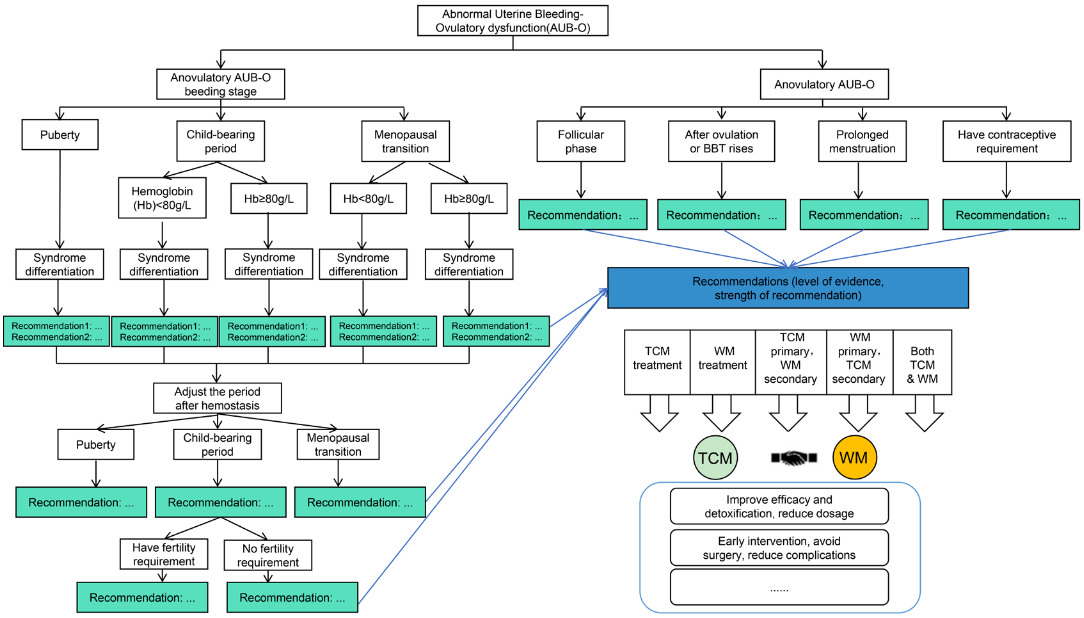

Fig. F1 This figure takes the abnormal uterine bleeding of ovulation disorders as an example to show how to organically integrate Western medicine disease and stage classification with Chinese medicine disease and syndrome types, so highlighting different clinical problems at different stages and providing corresponding TCM and Western medicine treatment recommendation strategies. In the process of formulating the guideline, the guideline makers should fully consider the corresponding relationship between TCM syndrome types and Western disease types from clinical practice, and put forward clinical questions according to the different stages, types and syndrome types of the diseases, culminating in the making of recommendations. This is the treatment strategy of integrated Chinese and Western medicine, thereby achieving the purposes of enhancing efficiency and reducing toxicity and complications, thus improving clinical efficacy.

Appendix G

Outline of the process and steps for developing clinical practice guidelines

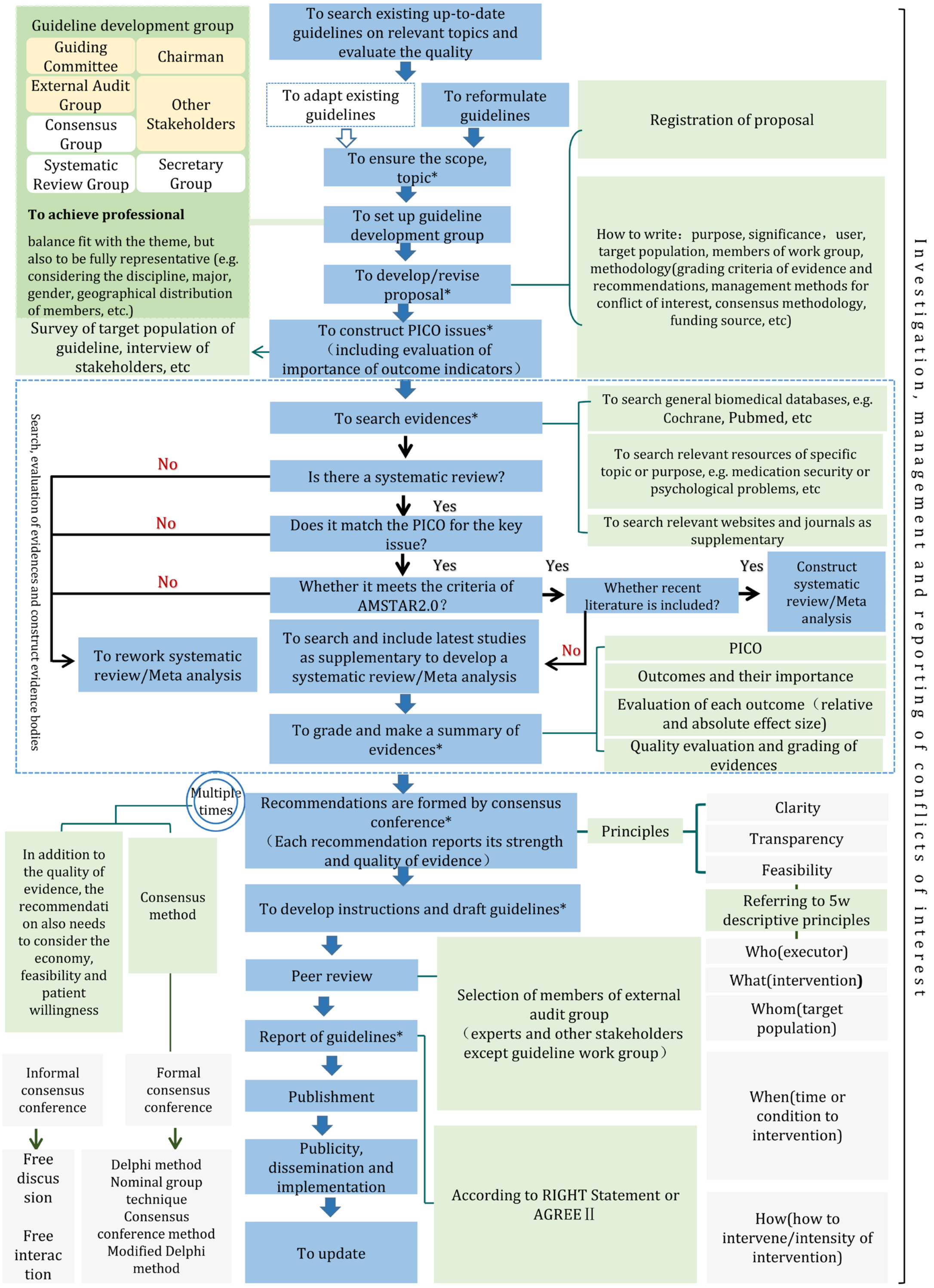

Fig.G1 Outline of the process and steps for developing clinical practice guidelines. Figure shows outline of the process and procedures for formulating clinical practice guidelines. *refers to the specific methodological content of the clinical practice guidelines for organic integration of traditional Chinese and Western medicine in this step,

corresponding to the standard text.

References

1. Chen KJ. [Two thoughts about the current integration of traditional Chinese and Western medicines]. J Tradit Chin Med.1978(6):3-5.
2. Chen KJ. [Recollection of 60 years of integrative medicine in China]. Chin J Integr Tradit West Med. 2017,37(2):140-142.
3. Zhao YN. [The future of integrated Chinese and Western medicine: from combination to integration]. Shanghai: Shanghai Science and Technology Press, 2016.
4. Institute of Medicine. Clinical practice guidelines we can trust. Washington, DC: National Academies Press, 2011.
5. Chen YL, Yang KH, Wang XQ, Kang DY, Zhan SY, Wang JY et al. [Guidelines for the development/revision of clinical practice guidelines in China (2022 edition)]. Nati Med J Chin. 2022,102(10):697-703.
6. Zhang W, Ma L, Deng HY. [Evidence evaluation tool for clinical randomized controlled trials of traditional Chinese medicine based on GRADE system]. Shanghai J Tradit Chin Med. 2022,56(4):6-13.
7. Chen ZG, Wang SC, Xu S, Luo H. [Methodological study on integrating the ancient and modern doctor's experience into evidence-based clinical practice guidelines of traditional Chinese medicine]. Chin J Tradit Chin Med Pharm. 2013,28(12):3609-11.
8. Chen W,Fang SN,Liu JP. [Recommendations for clinical evidence grading on traditional Chinese medicine based on evidence body]. Chin J Integr Tradit West Medi. 2019,39(3):358-64.
9. WHO. Handbook for guideline development. http://www.who.int/publications/guidelines/handbook_2nd_ed.pdf?ua=1. Accessed 16 Dec 2020.
10. NICE. Developing nice guidelines: the manual. https://www.nice.org.uk/process/pmg20/chapter/introduction-and-overview. Accessed 16 Dec 2020.
11. Wang J, Xiong XJ, Zhang LF. [Exploration of disease-syndrome combination mode and clinical application]. Chin J Integr Tradit West Med. 2012,32(3):297-9.
12. Wang L. [Complementary advantages of disease and syndrome differentiation-brief discussion on the understanding of Integrated Chinese and Western medicine]. Xinjiang J Tradit Chin Med. 2009,27(1):37-9.
13. Wang J, He QY, Li HX. [Exploration into methodology of integrative medicine and its development tendency]. Chin J Integr Tradit West Med. 2007, (06):565-7.
14. Wang CH, Feng Z, Yin ZW, Li S, Chen XM. [Development strategy of Chinese and Western integrative medicine under new circumstances]. Strategic Stu CAE. 2021,23(2):169-74.

15. Chen XM, Ni ZH, Liu YN, Xie YS, Sun W. [Guidelines for integrated traditional Chinese and Western medicine treatment of chronic renal failure]. Hebei J Tradit Chin Med. 2016,38(2):313-7.

16. Tong XL. [State target Medicine - the way of future development of traditional Chinese Medicine]. Chin J Integr Tradit West Med. 2021,41(1):16-8.
